# Self-Supporting Quasi-1D TaS_3_ Nanofiber Films with Dual Cationic/Anionic Redox for High-Performance Mg–Li Hybrid Ion Batteries

**DOI:** 10.1021/acsami.5c09460

**Published:** 2025-07-23

**Authors:** Pengcheng Jing, Atsushi Inoishi, Eiichi Kobayashi, Chengcheng Zhao, Peng Ren, Isaac Abrahams, Duncan H. Gregory

**Affiliations:** † WestCHEM, School of Chemistry, 3526 University of Glasgow, Joseph Black Building, Glasgow G12 8QQ, U.K.; ‡ Institute for Materials Chemistry and Engineering, 12923 Kyushu University, Kasuga-koen 6-1, Kasuga, Fukuoka 816-8580, Japan; § 133789 Kyushu Synchrotron Light Research Center, 8-7 Yayoigaoka, Tosu, Saga 841-0005, Japan; ⊥ Department of Chemistry, 4617 Queen Mary University of London, Mile End Road, London E1 4NS, U.K.

**Keywords:** magnesium−lithium hybrid ion batteries, tantalum trisulfide (TaS_3_), self-standing electrodes, nanofiber, cycling stability, mixed anionic and cationic redox

## Abstract

Magnesium–lithium hybrid ion batteries (MLIBs) offer a promising energy storage technology that combines the safety and dendrite-free plating/stripping of Mg anodes with the rapid Li^+^-dominated diffusion in cathode materials. However, for electrodes that undergo significant volume/structural changes during cycling, conventional slurry-cast fabrication often leads to microstructural degradation, active material detachment, and consequently, poor cycling stability and rapid capacity fading. Here, we report a self-standing, carbon- and binder-free tantalum trisulfide (TaS_3_) nano fibrous (NF) film, synthesized via a facile one-step physical vapor transport reaction that addresses these challenges through mechanistic innovations. Mechanistic investigations reveal that the TaS_3_ NF electrode undergoes dual cationic (Ta^5+^/Ta^3+^) and anionic (S_2_^2–^/S^2–^) redox reactions, accompanied by electrochemically induced phase transitions and *in situ* exfoliation. The dual redox couples provide a large number of Li^+^ ion storage sites, while the structural changes lead to fiber-level nanosizing, which in turn promotes fast (near) surface ion storage and pseudo capacitive behavior. Despite these significant transformations, the robust fibrous architecture retains structural integrity throughout prolonged cycling, as confirmed by *in* *operando* and *ex situ* characterization. This dual-redox, *in situ* exfoliation, and architecture-driven mechanism underpins the electrode’s exceptional cycling stability and high rate capability. As a result, the TaS_3_ NF electrode achieves a high reversible capacity of 178.5 mA h g^–1^ at 50 mA g^–1^, maintains 91.6% of reversible capacity after 100 cycles, and delivers 144.4 and 119.0 mA h g^–1^ at 500 and 1000 mA g^–1^, respectively, surpassing those of slurry-cast bulk TaS_3_ controls. Furthermore, the maintenance of a flexible film structure after extended cycling suggests potential applicability in next-generation wearable and structurally adaptive energy storage systems. These findings highlight the potential of self-standing, carbon- and binder-free film electrodes in advancing the cycling stability, energy density, and design versatility of MLIB systems and beyond.

## Graphical abstract

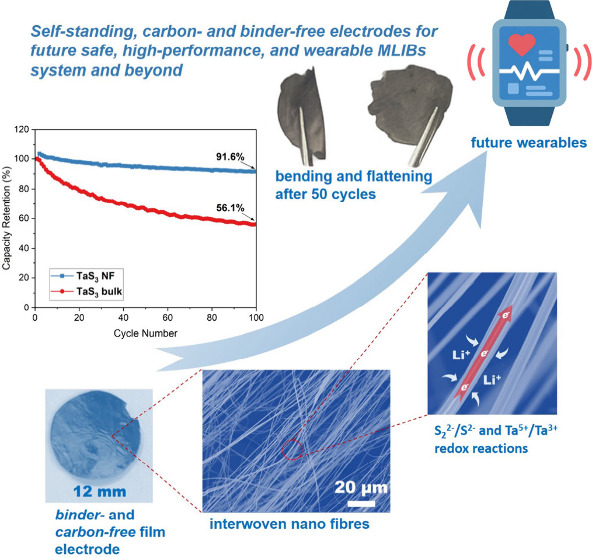

## 1 Introduction

The increasing global demand for sustainable and efficient energy storage solutions has driven the development of advanced rechargeable battery technologies.^1^ Among these, lithium-ion batteries (LIBs) have become the dominant choice due to their high energy density, long cycle life, and wide application in portable electronics and electric vehicles.^2^ Their exceptional energy density and extended cycle life have positioned them at the forefront of electrochemical energy storage solutions. However, the growing reliance on LIBs has raised significant concerns regarding the scarcity of lithium resources, fluctuating costs, and safety risks, including thermal runaway and flammable electrolytes.^3^ These limitations have prompted a reassessment of existing battery technologies and exploration of alternative systems that can meet future energy storage demands.

Magnesium-ion batteries (MIBs) have emerged as a promising alternative to LIBs, offering several inherent advantages. Magnesium metal anodes exhibit minimal dendrite formation during repeated plating and stripping processes in many high-performance electrolytes, significantly reducing safety risks associated with short circuits and fire hazards.^4^ This property enables the safe use of magnesium metal as an anode material in MIBs, offering a substantial capacity of 2205 mA h g^–1^, significantly surpassing the typical capacities of alternative anodes in LIBs such as graphite (*ca.* 372 mA h g^–1^).^5^ Furthermore, the abundance of magnesium and its low toxicity make it an attractive option for large-scale energy storage applications.^6^ Despite these benefits, the high charge density and strong polarizing nature of Mg^2+^ ions result in poor ion diffusion kinetics and low reversible magnesium storage capacities in most cathode materials, presenting a major challenge to the development of MIBs.^7^

To overcome these challenges, magnesium–lithium hybrid ion batteries (MLIBs) have been developed as a promising solution.^8^ MLIBs combine the safety and dendrite-free properties of Mg anodes with the rapid diffusion kinetics of Li^+^ ions in cathode materials. By introducing lithium salts (e.g., LiCl, LiBH_4_, and LiTFSI) into magnesium electrolytes, MLIBs enable reversible Li^+^ intercalation into cathode hosts while retaining the advantages of the magnesium anode. Notably, the thermodynamic incompatibility (−3.04 V for Li^+^/Li and −2.37 V for Mg^2+^/Mg vs SHE) prevents Li plating on Mg and underpins the operational stability of MLIBs, which has been confirmed by previous reports.^9^ This hybrid approach has demonstrated substantial performance improvements (compared to MIBs) in various intercalation-type cathode materials that have already proven effective in LIBs, such as polyanionic compounds (e.g., LiFePO_4_, LiTi_2_(PO_4_)_3_, and Li_3_V_2_(PO_4_)_3_; 100–150 mA h g^–1^)^10–12^ and transition metal oxides (TMOs; e.g., VO_2_ and TiO_2_; 150–200 mA h g^–1^).^13,14^ Transition metal chalcogenides (TMCs), with higher electrical conductivity than TMOs, have further been extensively explored. For instance, layered MoS_2_ has been employed in MLIBs and exhibited reversible capacity of *ca*. 210 mA h g^–1^ at a current density of 20 mA g^–1^.^15^ Layered VS_2_ was reported to exhibit a reversible capacity of 181 mA h g^–1^ at 50 mA g^–1^.^16^ Additionally, conversion-type TMC materials have been reported to exhibit considerable capacities; however, repeated conversion reactions often result in the agglomeration of metal particles and lithium/magnesium sulfides, leading to rapid capacity fading during long-term cycling.^17^

Despite these advancements, the conventional slurry-cast fabrication of cathodes presents certain possible limitations. The use of binders and conductive carbon introduces inactive components (20–30 wt %) into the electrode, reducing energy density and increasing the likelihood of parasitic side reactions.^18^ Additionally, for electrodes experiencing substantial volume and structural changes during repeated cycling, the adhesion between active materials and the conductive matrix is often insufficient.^19^ This can lead to active material detachment, inconsistent electrical conductivity, and ultimately rapid capacity degradation. Self-standing, flexible (SSF) electrodes have emerged as a feasible solution to these issues, particularly for electrodes subject to significant structural transformations, such as VS_4_.^20,21^ By eliminating the need for binders, conductive carbon, and current collector, SSF electrodes offer the potential for higher energy density. The interconnected framework in SSF electrodes facilitates reliable and long-lasting electrical and ionic transport while maintaining structural uniformity during discharge–charge cycles, mitigating significant structural changes and active material detachment, thereby enhancing cycling stability and capacity retention.^22^ Moreover, the inherent flexibility of SSF electrodes makes them particularly suitable for wearable energy storage devices, such as body-conformable health monitors, smart textiles, and other flexible electronics.^23^ These applications demand both high electrochemical performance and mechanical robustness to withstand repeated deformation during operation. As a result, SSF electrodes are increasingly recognized not only as one of the most effective solutions to the limitations of conventional electrodes undergoing substantial structural transformation, but also as a key enabler for advancing wearable energy storage technologies.

In recent years, (quasi-)­one-dimensional (1D) chain-like TMCs have garnered significant attention for their unique structural and electrochemical properties in rechargeable batteries. Vanadium pentasulfide (VS_4_) is a notable example that has been extensively studied in LIBs, MIBs and MLIBs, among others.^24,25^ In VS_4_, redox-active S_2_^2–^ anions, in conjunction with V^4+^ cations, participate in a dual anionic/cationic redox process, enabling multielectron transfer and delivering extra capacity. This mechanism contrasts with layered transition metal dichalcogenides containing only nonreducible S^2–^ anions. When applied in MLIBs, VS_4_ achieves notable capacities ranging from 300 mA h g^–1^ to 400 mA h g^–1^,^21,26,27^ yet suffers from pronounced capacity decay during initial cycles, likely due to structural degradation and active material detachment. Beyond VS_4_, however, dual redox-active (quasi-)­1D TMCs remain largely underexplored in MLIBs, highlighting the importance of investigating alternative materials capable of harnessing both cationic and anionic redox processes in a stable and structural robust framework. SSF tantalum trisulfide (TaS_3_), a typical quasi-1D chain-like TMC featuring both S_2_^2–^ and S^2–^ in its structure, has been previously synthesized via a physical vapor transport (PVT) method and studied as an electrode material for LIBs.^28,29^ Despite the scarcity of Ta, its use in TaS_3_ film electrodes is justified by the high sustainability. Unlike other electrode materials that may require complex recycling processes, self-standing TaS_3_ films can be easily recycled and regenerated due to their simple structure, free of complex additives or binders, making them an environmentally friendly option for battery applications.

In this study, we present, for the first time, the application of self-standing, flexible, nano fibrous TaS_3_ films as cathode materials for MLIBs. These films are composed of interwoven ultralong nanofibers, ranging from 100 nm to several hundred nanometers in lateral dimensions, which are formed by stacking smaller fibers. This unique architecture ensures stable electrical conductivity, efficient ion transport, and mechanical robustness during cycling. Electrochemical evaluations reveal that TaS_3_ nanofiber (NF) electrodes outperform conventional slurry-cast counterparts, delivering a high reversible capacity of 178.5 mA h g^–1^ at 50 mA g^–1^, robust cycling stability with 91.6% capacity retention over 100 cycles, and excellent rate capability. Mechanistic studies (via *ex situ* scanning electron microscopy (SEM) and *in operando* powder X-ray diffraction (PXRD)) demonstrate that the fibrous microstructure remains intact, despite significant phase/structural changes, exfoliation and *in situ* nanosizing during cycling. The high Li^+^-dominated capacity arises from mixed cationic (Ta^5+^/Ta^3+^) and anionic (S_2_^2–^/S^2–^) redox reactions, as confirmed by *ex situ* X-ray photoelectron spectroscopy (XPS), X-ray absorption spectroscopy (XAS), and Raman spectroscopy. Furthermore, the robust flexibility of TaS_3_ NF films makes them particularly suitable for integration into wearable and flexible electronic devices, where mechanical durability and lightweight properties are essential. These findings highlight the potential of self-standing, carbon- and binder-free film electrodes to address the limitations of conventional electrodes and pave the way for future high-energy-density, cycling-stable, and wearable MLIB systems and beyond.

## 2 Experimental Section

All experiments outlined below were conducted at room temperature (RT), unless explicitly stated otherwise.

### 2.1 Synthesis of TaS_3_ Nanofibers (NFs) and Bulk TaS_3_

The synthesis of TaS_3_ NFs was conducted via a modified physical vapor transport (PVT) reaction, using a custom-designed fused-silica tube reactor (Figure S1a), similar to one described in our previous work.^30^ The reactor tube (250 mm in length, 12 mm in inner diameter) featured a neck positioned 120 mm from the base, allowing the spatial separation of tantalum powder (Aldrich, 99.5%) placed at the bottom and sulfur pellets (Sigma-Aldrich, 99.98%) positioned above the neck. This configuration serves two critical purposes: (i) it prevents premature contact between molten sulfur and solid tantalum, which could otherwise trigger uncontrolled side reactions and hinder the anisotropic growth of TaS_3_ NFs; (ii) it ensures that tantalum is only exposed to sulfur vapor during heating, thereby facilitating uniform vapor–solid reaction and the formation of well-defined fibrous products. Compared to conventional chemical vapor transport (CVT) methods, this PVT approach does not require any transport agents and minimizes the formation of byproducts. In addition, the method is readily scalable: by appropriately adjusting the inner diameter and pressure-bearing capacity of the silica tube, as well as the quantity of starting materials, large-scale synthesis can be achieved without compromising product quality. These features make this approach particularly attractive for the fabrication of self-standing electrode films toward practical applications.

Specifically, 0.9 g of tantalum powder was transferred into the tube through the neck using a small funnel, followed by placing the sulfur pellet near the neck. Subsequently, the tube was evacuated and flame-sealed under a still vacuum of 3 × 10^–4^ mbar. The sealed tube was then placed horizontally in a box furnace (Brothers Box Furnace, BR-12N-5) and heated to 550 °C at a rate of 200 °C h^–1^ for a dwell time of 66 h. During this heating process, the sulfur pellet melts and then evaporates, or sublimes into sulfur gas, which transports to the bottom of the tube and reacts with the Ta powder to form the TaS_3_ NF film. After the reaction, the tube was allowed to naturally cool to RT. A photograph of the quartz tube containing the TaS_3_ NFs after heating and cooling to room temperature is provided in Figure S1b. The product consists of the fiber aggregates located in the upper region and the bulk condensed particles located at the bottom (Figure S1c).

The fiber aggregates can be easily separated and spread using tweezers and a doctor blade. As illustrated in Figure S1d, the as-collected fiber aggregates, resembling a “trouser leg”, can be made with a straight cut along the length of the “leg” (simplified as a cylindrical film) using sharp scissors. Subsequently, the cut fiber aggregates can be spread and flattened using a doctor blade, forming the flat film that can be cut into film discs as electrodes for MLIBs. These self-standing TaS_3_ films are soft and can undergo folding up to 180° without being ruptured, indicating good structural flexibility (Figure S2).

### 2.2 Synthesis of Cubic Li_2_TaS_3_

Cubic Li_2_TaS_3_ was synthesized for the first time by ball milling. Initially, TaS_2_ was synthesized from a mixed powder of 0.9 g of Ta and 0.32 g of S using a similar method as described for the TaS_3_ synthesis, except for the absence of a neck on the quartz tube. Subsequently, 0.842 g of the as-synthesized TaS_2_ powder and 0.158 g of Li_2_S powder (Sigma-Aldrich, 99.98%) were hand-mixed and sealed in a 50 mL tungsten carbide (WC) jar in an Ar-filled glovebox. Additionally, 10 WC balls (10 mm diameter, 7.6 g per ball) were placed in the jar to ensure a ball-to-powder ratio of 76:1. A Retsch, PM 100 planetary ball mill was employed for milling, with parameters set to 510 rpm for 60 h, with intervals of 2 min between 5 min of reverse rotations. After the mechanochemical synthesis, a dark brown powder was collected and stored in the glovebox.

### 2.3 Material Characterization

As-synthesized air-stable samples were characterized in air atmosphere, whereas the air-sensitive cycled samples were handled and measured under vacuum or inert gas (Ar or N_2_) atmosphere using homemade vessels, such as flame-sealed capillaries and airtight domed holders.

PXRD patterns were acquired in Bragg–Brentano geometry (flat plate, reflection) over a 2θ range of 5°–70° with a step size of 0.0175° and a scan rate of 0.083° s^–1^ on a Rigaku MiniFlex diffractometer equipped with a Cu K_α_ (λ = 0.154 nm) X-ray source operated at 40 kV and 40 mA and in transmission geometry (capillary, 5°–70°, 0.0131°, 0.016° s^–1^) on a PANalytical Empyrean diffractometer with an unmonochromated Cu K_α_ X-ray radiation operated at 45 kV and 40 mA. *In operando* PXRD patterns were collected in Bragg–Brentano geometry (flat plate, reflection) between 5°–65° 2θ with a step size of 0.0263° at a scan rate of 0.074° s^–1^ on the PANalytical Empyrean diffractometer. For *in* *operando* PXRD experiments, a homemade CR2032 coin cell with a 7 μm-thick Kapton window (8 mm diameter, sealed with glue and PVDF) at the center of the positive case was employed.

Raman spectra were collected at RT using a LabRAM HR spectrometer equipped with a green laser with a wavelength of 532 nm. The as-synthesized samples were prepared and measured in air by pressing the TaS_3_ film and powder onto glass slides. The cycled samples were sealed in Ar by flame. TG-DSC measurement was performed using a TA Instruments SDT Q600 instrument, ramping from RT to 650 °C at a rate of 10 °C min^–1^ in a 2% O_2_/98% Ar atmosphere (100 mL min^–1^).

Constituent species and their oxidation states were probed by X-ray photon spectroscopy (XPS) using Thermo Scientific Nexsa and Kratos AXIS Supra+ X-ray photoelectron spectrometers, both equipped with an Al Kα X-ray source. All high resolution XP spectra were analyzed and curve fitted according to Conny and Powell, typically employing dual Gaussian–Lorentzian functions to obtain precise binding energies.^31,32^ X-ray absorption spectroscopy (XAS) measurements for the S K-edges (2440 to 2500 eV) were performed at the BL2A beamline of the UVSOR Synchrotron Facility, Institute for Molecular Science (Japan). The spectra were collected in total electron yield mode. SEM-EDS experiments were performed on a TESCAN CLARA microscope equipped with an Oxford Instruments UltimMax 65 energy dispersive X-ray spectrometer at 15 kV. The TaS_3_ NFs and bulk powdered specimens were prepared by adhering a small piece of TaS_3_ NF film or scattering a small amount of TaS_3_ bulk powder onto conductive carbon tape in air and coating with a thin layer (*ca*. 10 nm of thickness) of gold via plasma sputtering. Electrochemically cycled electrodes were prepared in the glovebox without gold coating and sealed in a 14 mL vial before being promptly transferred to the SEM chamber, with an air exposure time of less than 10 s. TEM imaging was conducted on a FEI Tecnai G2 F30 Microscope (300 kV). The TEM specimens were prepared by dispersing approximately 1 mg of the as-made TaS_3_ NFs in 1 mL absolute ethanol in an ultrasonic bath for 5–10 min. Subsequently, one drop of the dispersion was deposited onto an ultrathin carbon-coated Cu TEM grid and air-dried for an hour.

### 2.4 Electrochemical Measurements

The electrochemical performance of both TaS_3_ NF and bulk TaS_3_ electrodes was assessed in CR2032 type coin cells. Magnesium metal foil pieces (0.2 mm thickness, diameter 15 mm, 99.5%, sourced from Huabei Magnesium Processing Plant) were used as anodes. As-synthesized TaS_3_ NFs were directly used as cathodes. For bulk TaS_3_ electrodes, slurries were prepared by blending 0.07 g of active material, 0.02 g of conductive carbon (carbon black, 99%, Alfa Aesar), and 0.01 g of polyvinyldifluorine (PVDF, 98%, average molecular weight ∼ 534,000, Sigma-Aldrich) binder in *ca.* 0.5 mL of *N*-methyl pyrrolidone (anhydrous, 99.5%, Sigma-Aldrich). The resulting slurries were coated onto carbon paper chips (12 mm diameter, 0.19 mm thickness, areal density of 0.44 g cm^–2^, Saibo Electrochemistry) and dried under vacuum (0.1 mbar) in a drying oven at 60 °C overnight. The typical mass of the self-standing TaS_3_ NFs or slurry-cast bulk TaS_3_ on the carbon paper current collector was around 1–2 mg, or 0.88–1.77 mg cm^–2^. In an Ar-filled MBraun LabStar glovebox (O_2_ and H_2_O < 0.5 ppm), 10.0 mL solution of 0.4 M “all-phenyl complex” (APC, MIB electrolyte) electrolyte was prepared by dropwise addition of 4.0 mL of a solution of phenyl magnesium chloride (PhMgCl, 2.0 M in tetrahydrofuran (THF), Sigma-Aldrich) into 0.534 g of AlCl_3_ (ultra dry, 99.99%, Thermo Fisher Scientific) in 6 mL of THF (≥99.9%, anhydrous, inhibitor-free, Sigma-Aldrich) under magnetic stirring. Additionally, a 2.0 mL 1 M LiCl-APC (MLIB electrolyte) solution was prepared by adding 0.085 g of LiCl (≥99.9%, Aldrich) into 2.0 mL of 0.4 M APC, followed by stirring overnight in the glovebox. The electrolyte used for the assembly of comparative LIBs was 1.0 M LiPF_6_ in EC/DEC = 50/50 (v/v) (battery grade, Sigma-Aldrich). The volume of electrolyte used for the assembly of coin cells was around 90 μL.

Discharge and charge experiments, as well as galvanostatic intermittent titration technique (GITT) experiments, were conducted using a LAND CT2001A battery test system over a voltage range of 0.4–2.2 V. For GITT experiments, the test batteries were discharged for 600 s at a current density of 25 mA g^–1^, followed by a relaxation period (no current applied) of 1200 s. These discharging/relaxation or charging/relaxation steps were repeated until the discharging limit of 0.4 V or charging limit of 2.2 V was reached. The GITT curve data and details on how they were used to calculate diffusion coefficients are provided in Equation S4 and Figure S19. Cyclic voltammetry (CV) and electrochemical impedance spectroscopy (EIS) measurements were performed using a PalmSens4 potentiostat at RT. CV experiments were conducted over a voltage range of 0.4–2.2 V at scan rates of 0.1–0.5 mV s^–1^. Scans began from the open-circuit voltage down to the low-voltage cutoff, followed by a sweep up to the high-voltage cutoff. EIS experiments were performed over a frequency range of 100 000 to 0.01 Hz with a potential amplitude of 10 mV. The obtained Nyquist plots were fitted using AfterMath software.^33^

## 3 Results and Discussion

The PXRD patterns of TaS_3_ NFs and bulk TaS_3_ are presented in Figure S3. It can be observed that peaks in the pattern of TaS_3_ NFs can be mainly assigned to an *m*_1_-TaS_3_ monoclinic phase with space group *C*2/*m*,^34^ while the pattern of bulk TaS_3_, with quite a few reflections absent, may be considered an orthorhombic structure (*o*-TaS_3_; PDF - 18–1313; space group of *C*222_1_). However, the assignment of bulk TaS_3_ is not conclusive due to the lack of detailed crystal structure information for this orthorhombic phase in the literature. Previous studies indicate that TaS_3_ NFs comprise mainly of the *m*_1_-phase with a minority of a second monoclinic phase *m*_2_ (*m*_2_-phase) present.^34^ Therefore, Rietveld refinement of the PXRD data (transmission geometry, capillary mode) was performed based on the above two-phase mode to achieve compositional and structural parameters for the two components in the as-synthesized TaS_3_ NF product. As shown in Figure 1a and Tables S1–3, the refinement results reveal a mixture of *m*_1_- and *m*_2_-phases in the TaS_3_ NFs product, accounting for 96.6(5) wt % and 3.4(2) wt %, respectively. The refined crystal parameters for *m*_1_-phase, *a* = 19.9127(9) Å, *b* = 3.3382(1) Å, *c* = 15.1685(3) Å, and β = 112.463(5)°, are comparable to published values.^34^ The crystal information on the *m*_2_-phase is provided in Tables S2–3. Rietveld refinement for bulk TaS_3_ was also attempted using the *m*_1_-phase crystal structure, and the corresponding results are provided in Figure S4 and Table S4. Similar to the initial flat plat PXRD analysis, it was observed that the refinement could not resolve some obvious peaks for the *m*_1_-TaS_3_ phase, and the refined results show a greater deviation to the *m*_1_-TaS_3_ model when compared to those of TaS_3_ NFs. From the overall refinement profile, however, it appears that bulk TaS_3_ exhibits a structure similar to TaS_3_ NFs but with notably higher symmetry, probably due to the slightly different chain arrangements in their structures. The crystal structure for TaS_3_ NFs is shown in Figure 1b-d. TaS_3_ (using the major *m*_1_-phase in NFs as an example) consists of pseudolayers separated by a van der Waals (vdW) gap. These pseudolayers are formed from the arrangement and weak bonding of infinite 1D [TaS_6_]_*n*_ chains (which extend along the *b*-axis via S–Ta–S polar covalent bonds) along the *c*-axis. The interlayer spacing of the (002) reflection is *ca*. 9.2 Å for the dominant *m*_1_-phase in the TaS_3_ NF material, as derived from the Rietveld refinement.

**Figure 1:**
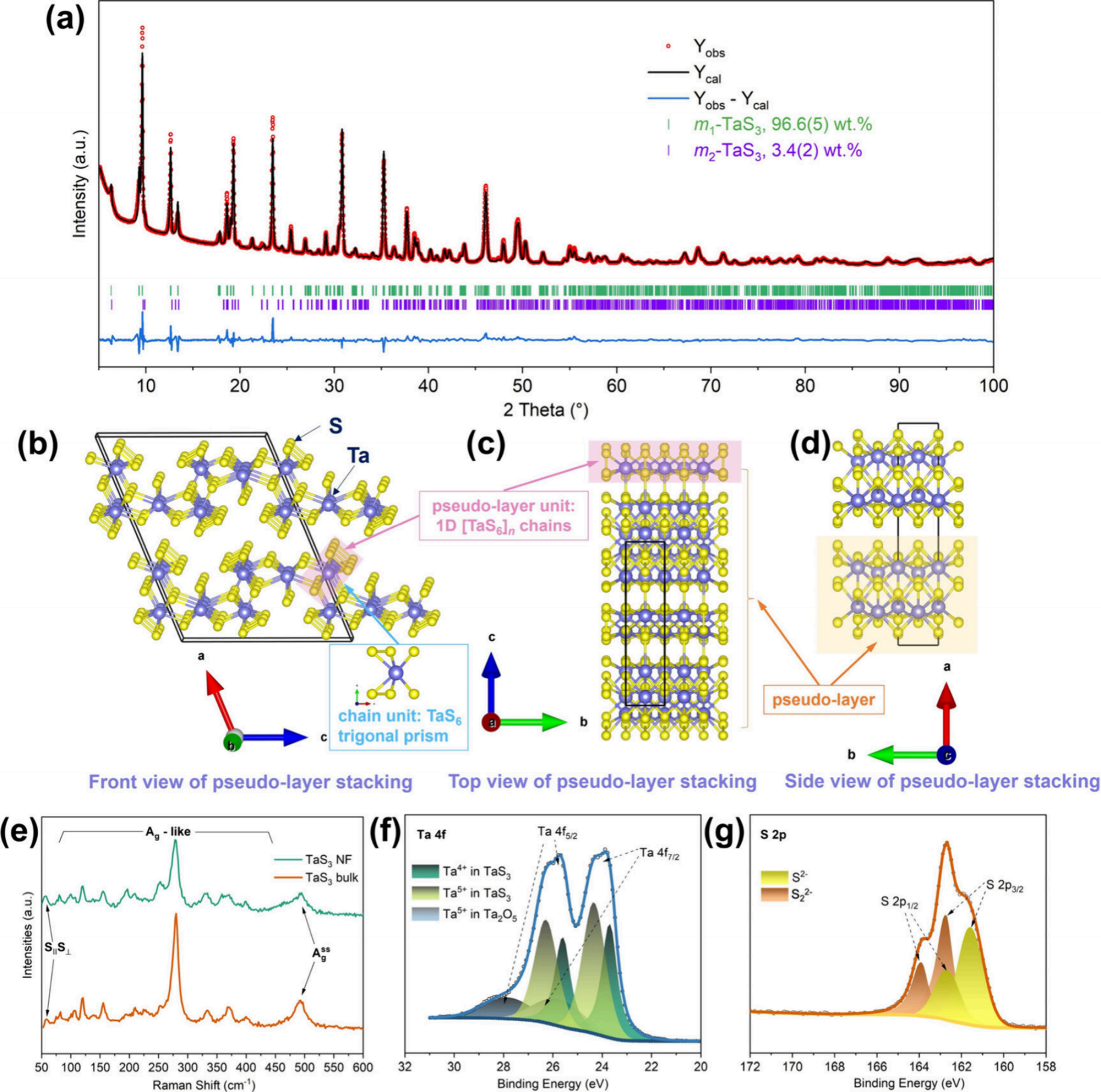
(a) Fitted PXRD diffraction profile for TaS_3_ NFs (transmission geometry, capillary mode). Crystal structure of TaS_3_ NF showing projections down the (b) *b*-axis, (c) *a*-axis, and (d) *c*-axis, with unit cell shown, as visualized by Vesta software.^38^ (e) Raman spectra of as-made TaS_3_ NFs and bulk TaS_3_. High-resolution XPS spectra of the: (f) Ta 4f and (g) S 2p regions for TaS_3_ NFs.

Raman spectra of the as-prepared TaS_3_ NFs (*m*-phase) and bulk TaS_3_ (possibly *o*-phase) are presented in Figure 1e. The signals in both spectra are consistent with those of TaS_3_ NFs reported and theoretically calculated by Mayorga-Martinez *et al*.,^34^ and are comparable to the vibrational modes of TaS_3_ NFs investigated by Wu *et al*.^35^ The peak at approximately 58 cm^–1^ can be attributed to two-shear (*S*) eigenmodes, namely, interchain displacement parallel (S_∥_) and perpendicular/nonparallel (S_⊥_) to the Ta-chain. The peaks ranging from 60 cm^–1^ to 425 cm^–1^ are likely due to A_g_-like vibrational modes which are yet to be determined accurately, while the signal at approximately 495 cm^–1^ is ascribed to A_g_^S–S^ modes. The slightly higher intensity of the peak at 253 cm^–1^ is attributable to the presence of the *m*_2_-phase in the TaS_3_ NFs.^34^ Note that Raman spectroscopic studies of TaS_3_ remain very limited and nonsystematic, thus the above discussion should be considered instructive instead of conclusive.

XPS was employed to investigate the surface chemical states of the Ta and S elements in TaS_3_ NF. Six peaks are observed in the fitted Ta 4f high-resolution spectrum (Figure 1f). The doublet peaks at *ca.* 23.7 and 25.6 eV are ascribed to the Ta 4f_7/2_ and Ta 4f_5/2_ transitions, respectively, and match well with the characteristic binding energies of Ta^4+^ (those coordinated with disulfide anions) in tantalum chalcogenides.^34^ Given that Ta in TaS_3_ has a formal (or average) valence state of around +4.67,^34^ the peaks at *ca.* 24.3 and 26.3 eV can be attributed to the Ta^5+^ (those coordinated with sulfide anions) of TaS_3_. Indeed, by calculating the atomic percentages of Ta^5+^ (*ca.* 58.5%) and Ta^4+^ (*ca.* 41.5%) species of TaS_3_ from the Ta 4f spectrum, a mean valence state of +4.59 is obtained, which closely aligns with the theoretical value. The last pair of peaks at *ca.* 25.9 and 28.0 eV are typical for Ta^5+^ in Ta_2_O_5_.^36^ It is not surprising to witness the surface oxidation of the TaS_3_ NFs given their air sensitivity and their tendency to release H_2_S, for example, when exposed to air. This surface oxidation is further supported by multiple lines of evidence: (a) the appearance of Ta–O signals in both the Ta 4f and O 1s spectra after Ar^+^ etching (Figure S5a, b), indicating the presence of tantalum oxide species at or near the surface; (b) the identification of a monoclinic Ta_2_O_5_ layer on the fiber surface, as revealed by HRTEM analysis (Figure 2f); and (c) the absence of any Ta–O phase features in the PXRD pattern and Raman spectra, along with the uniform fibrous morphology observed in SEM images (Figure 2a, b), which shows no evidence of secondary oxide phases. The high-resolution spectrum in the S 2p region shows four peaks after data fitting (Figure 1g). The paired peaks at *ca.* 162.7 and 163.9 eV, corresponding to S 2p_3/2_ and S 2p_1/2_, can be attributed to S_2_^2–^ species.^26,27^ Meanwhile, the second set of peaks at *ca.* 161.6 and 162.7 eV is ascribed to S^2–^ in TaS_3_.^37^ The total negative charge from S_2_^2–^ and S^2–^ species (−4.59) balances the positive charge of Ta ions in TaS_3_, in accordance with charge neutrality principles. Furthermore, the Ta 4f and S 2p spectra of bulk TaS_3_ (Figure S5c, d) closely resemble those of TaS_3_ NF, confirming their comparable chemical environments.

**Figure 2:**
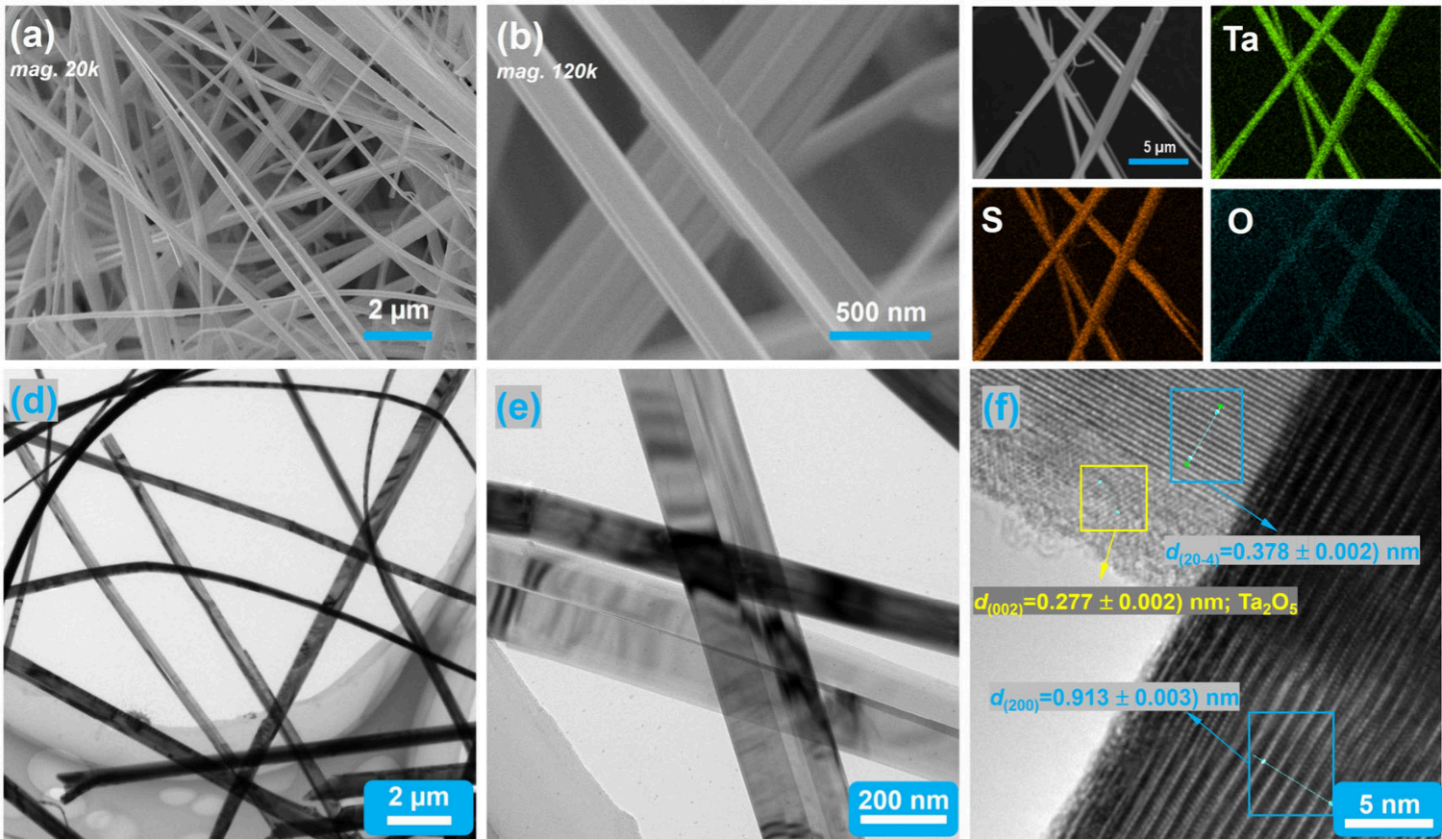
Results of electron microscopy characterization on the as-prepared TaS_3_ NFs displaying: (a) low-magnification (×*20k*) and (b) high-magnification (×*120k*) SEM images; (c) SEM-EDS elemental maps for Ta, S, and O, respectively; (d) low-magnification, (e) high-magnification TEM, and (f) HRTEM images.

The morphology and composition of TaS_3_ NFs and bulk TaS_3_ were characterized using SEM-EDS and TEM instruments. In Figures 2a, b, SEM images at low and high magnifications reveal that the former sample exhibits a network structure composed of numerous ultralong fibers with length of a few centimeters (also see Figure S1c) and lateral size of less than 1 μm. Looking closer, each micron-sized fiber appears to be made up of bundles of nanofibers with diameters spanning 100 to 300 nm. In contrast, bulk TaS_3_ consists mainly of micron-sized particles ranging from several micrometres to tens of micrometres, although the enlarged SEM image shows densely packed short needle-shaped crystallites (Figure S6). An SEM image and the corresponding elemental maps for Ta, S, and O in fibers shown in Figure 2c, illustrate uniform and homogeneous distributions of the constituent elements. The EDS spectrum (Figure S7) indicates an S:Ta mole ratio of 2.67:1, slightly lower than the stoichiometric value of 3:1. This deviation could be attributed to surface oxidation and/or inaccuracy in the quantification of Ta due to the lack of suitable elemental EDS standards for this element. Previous reports have noted even lower S-to-Ta ratios (of ∼2) in TaS_3_ due to the absence of standardization.^34^ To obtain a more accurate S/Ta ratio on a bulk scale, a TGA experiment was conducted in an O_2_/Ar atmosphere, and the calculation details and results are shown in Figure S8. The S/Ta ratio calculated through TGA is 2.91 ± 0.02, corresponding very well to the stoichiometry of TaS_3_. The slight sulfur deficiency may be related to the presence of certain amount of sulfur vacancies within TaS_3_ NF, which, well documented in the literature, facilitate ion diffusion and provide additional sites for ion storage.^39,40^ TEM images in Figure 2d, e exhibit numerous cross sections of the nanofibers, confirming their lateral dimensions (typically 100–250 nm) in good accordance with the SEM results. The HRTEM image further reveals lattice fringes in single nanofibers (Figure 2f). Measurements of different lattice fringe distances were performed using the DigitalMicrograph software^41^ (Figure S9), yielding *d-*spacings of 9.13(3) Å for the (200) crystal plane and 3.78(2) Å for the (20 ®4) plane, both associated with the *m*_1_ and/or *m*_2_ phase identified in the PXRD pattern. Additionally, a third set of lattice fringes, observable near the periphery of the fiber region exposing the (20 ®4) plane, was indexed to the (002) planes of monoclinic Ta_2_O_5_, corroborating the presence of surface oxidation as previously inferred from the XPS analysis.

The electrochemical performance of the as-synthesized TaS_3_ NF electrodes in MLIBs was determined and compared with slurry-cast electrodes prepared from bulk TaS_3_. Two key aspects are assessed: (a) the significance of the LiCl additive to the electrochemical properties of the TaS_3_ electrode and (b) the effect of the flexible fibrous morphology on the performance of the electrodes. Initially, the impact of LiCl addition to the “all phenyl complex” (APC) electrolyte on the performance of the self-standing nanofibrous TaS_3_ electrode was investigated using a cyclic voltammetry (CV) approach. CV curves of the TaS_3_ NF electrode in LiAPC and APC electrolytes were obtained at a scan rate of 0.1 mV s^–1^. In pure APC electrolyte, no electrochemical redox peaks are observed in the CV curves of the TaS_3_ NF electrode (Figure S10a), indicating negligible magnesium ion intercalation. However, as depicted in Figure 3a, with the presence of LiCl in the electrolyte, significant cathodic/anodic redox peaks emerge at *ca.* 0.74 V/ (1.09, 1.61, and 1.91 V) in the first CV scan, indicating the intercalation/deintercalation of Li^+^ and possibly Mg^2+^ ions. The second CV curve exhibits a broadened and weakened cathodic peak at higher voltage compared to the first cycle which suggests an irreversible change during the first cycle. Subsequent scans show cathodic peaks, corresponding to the reduction of S_2_^2–^ and Ta^5+^ ions (See mechanistic analysis in coming sections), shift to progressively higher voltages (*ca*. 1.45 and 1.04 V, respectively) and appear to remain, with stabilized anodic peaks at *ca*. 1.15, 1.58, and 1.96 V, indicating improved electrochemical properties and stable, reversible redox reactions. CV curves of the TaS_3_ NF electrode in an LIB and the bulk TaS_3_ electrode in an MLIB were also obtained for comparison (Figure S11). Both electrodes in MLIBs exhibit similar electrochemical behavior to the TaS_3_ NF electrode in LIBs, indicating the intercalation of Li^+^ cations as the primary charge carriers. The initial four (dis)­charge curves of the TaS_3_ NF electrode at a current density of 50 mA g^–1^ are presented in Figure 3b. A plateau at approximately 0.85 V contributes to a first discharge capacity of *ca*. 197 mA h g^–1^, while the first charge curve exhibits two slopes in the ranges of *ca*. 1.1–1.3 V and 1.6–1.8 V, respectively, and a plateau at *ca*. 1.94 V, suggesting an irreversible change during the initial cycle. The first charge capacity is 179 mA h g^–1^, resulting in a high initial Coulombic efficiency of *ca*. 91%. Subsequent (dis)­charge cycles exhibit similar electrochemical behavior, with improved discharge voltage and reduced voltage hysteresis. Conversely, when galvanostatic cycling was performed in cells with a pure APC electrolyte, the TaS_3_ NF electrode showed a discharge capacity of approximately 5.6 mA h g^–1^ in the first cycle and even less in subsequent cycles (Figure S10b). These observations align with the CV results discussed above. For comparison, the initial four (dis)­charge curves of the TaS_3_ NF electrode in an LIB and of the bulk TaS_3_ electrode in an MLIB are also provided (Figure S12). The presented (dis)­charge curves closely resemble those of the TaS_3_ NF electrode in an MLIB, indicating that the charge storage capacity of the TaS_3_ electrode materials in MLIB can be primarily attributed to Li^+^ cations.

**Figure 3:**
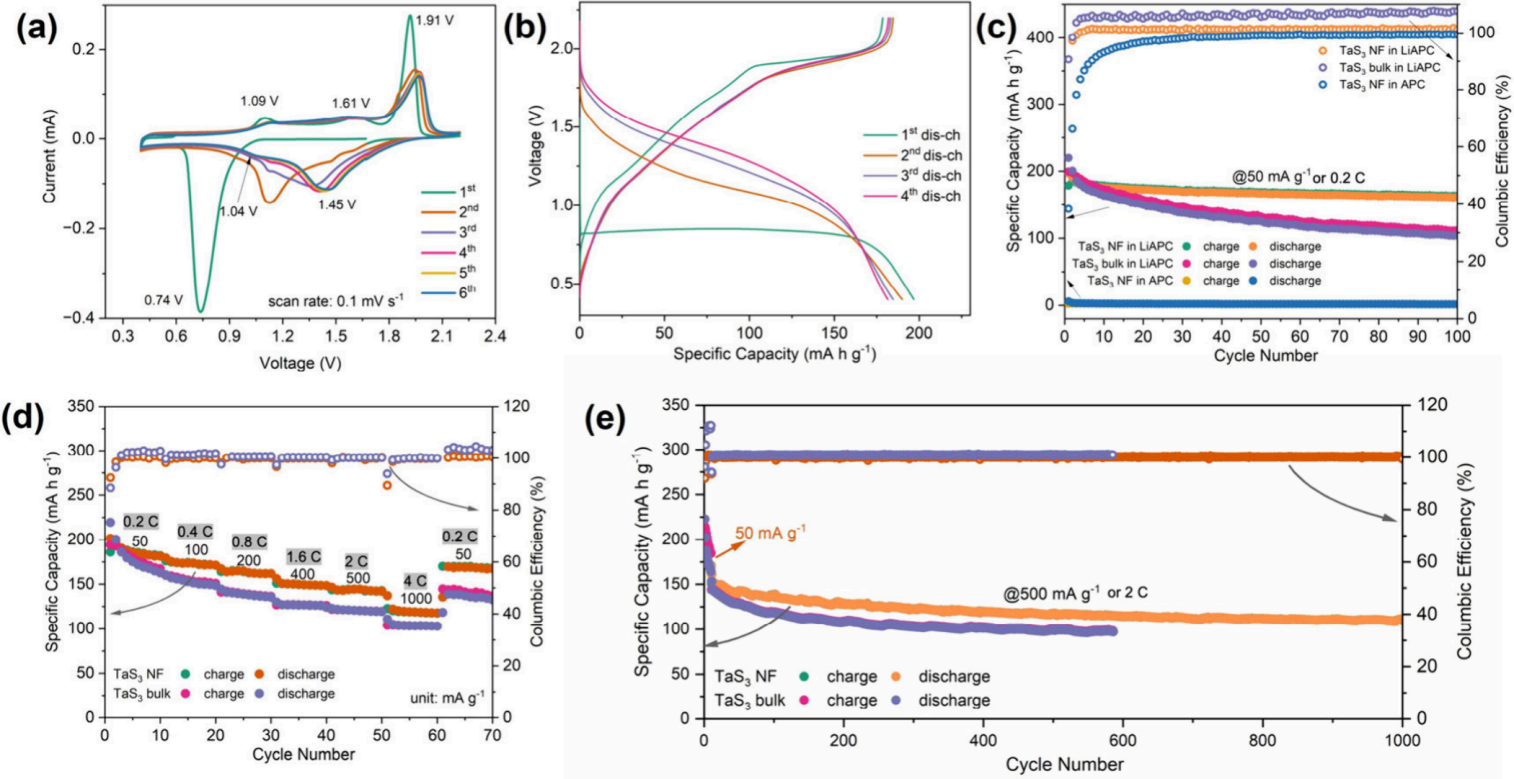
Galvanostatic cycling performance of TaS_3_ NF and bulk TaS_3_ electrodes: (a) Initial six CV curves of the self-standing TaS_3_ NF electrodes at a scan rate of 0.1 mV s^–1^ between 0.4 and 2.2 V and (b) the initial four (dis)­charge curves of the same electrode at a current density of 50 mA g^–1^ in an MLIB. (c) Cycling performance at a current density of 50 mA g^–1^ (TaS_3_ NF in LiAPC and APC, TaS_3_ bulk in LiAPC), (d) variable current densities over 70 cycles (TaS_3_ NF and TaS_3_ bulk in LiAPC), and (e) at a high current density of 500 mA g^–1^ to evaluate long-term cycling performance (TaS_3_ NF and TaS_3_ bulk in LiAPC).

The investigation then delved into the impact of the flexible fibrous morphology on the performance of the TaS_3_ electrode in MLIBs. Direct fabrication of a slurry-cast electrode using the TaS_3_ NF film was not feasible (see Note S1 in the Supporting Information) due to its mechanical integrity. Instead, bulk TaS_3_ with a similar crystal structure was used as a control material. 1C is defined as 250 mA g^–1^, based on the theoretical capacity under the assumption of full involvement of the Ta^5+^/Ta^3+^ cationic and S­(-I)/S­(-II) anionic redox couples, as supported by XPS and XAS analysis (Figure 5). Figure 3c illustrates the cyclic performance of the TaS_3_ NF electrode compared to its bulk material counterpart at a current density of 50 mA g^–1^ or 0.2 C. It is evident from the graph that TaS_3_ NF exhibits steady cycling behavior, retaining an excellent 91.6% of its reversible charge capacity after 100 cycles (*ca*. 163.5 mA h g^–1^ out of 178.5 mA h g^–1^), while the capacity of the bulk TaS_3_ electrode significantly diminishes to only 56.1% (111.7 mA h g^–1^). These results underscore the enhanced electrochemical performance derived from the uniform and interconnected nanofibrous morphology of the self-standing and binder-free TaS_3_ electrode material. It is worth noting that the subtle differences in the crystal structure between TaS_3_ NF and bulk forms may also contribute to the observed variations in electrochemical performance, albeit to a lesser extent. The integrated structure is expected to prevent material exfoliation and detachment from the matrix, thereby reducing potential inactive (“dead”) electrode parts during repeated discharge and charge cycles and maintaining a stable Li^+^ cation/electron transport network within the nanofibers. Indeed, it has been widely reported that free-standing interconnected electrode materials exhibit superior performance compared to conventional slurry-prepared electrodes.^22,42^ However, most of these active materials in free-standing electrodes are attached to carbon nanofibers, which inevitably compromises capacity and energy density. An example of a chalcogenide electrode that does not rely on a carbon matrix is self-standing, 3-dimensional, and interconnected porous MoS_2_, which demonstrates superior performance in LIBs compared to other MoS_2_ electrode materials blended with carbon and binders.^43,44^ As in the CV measurements, when using *unmodified* APC electrolyte, TaS_3_ could only deliver a negligible capacity, emphasizing the crucial role of LiCl as a provider of Li^+^ in the functioning of TaS_3_ electrode materials.

The TaS_3_ NF electrode also demonstrates robust rate capability compared to bulk TaS_3_ (Figure 3d). With continuously increasing current densities of 50 mA g^–1^ (0.2 C), 100 mA g^–1^ (0.4C), 200 mA g^–1^ (0.8C), 400 mA g^–1^ (1.6C), 500 mA g^–1^ (2C), and 1000 mA g^–1^ (4C) every ten cycles, TaS_3_ NF maintains high discharge capacities of *ca.* 185.5 mA h g^–1^, 173.7 mA h g^–1^, 164.9 mA h g^–1^, 150.0 mA h g^–1^, 144.4 mA h g^–1^, and 119.0 mA h g^–1^, respectively. However, the bulk TaS_3_ electrode delivers lower capacities of 120.7 mA h g^–1^ and 103.3 mA h g^–1^ at the higher rates of 500 mA g^–1^ (2C) and 1000 mA g^–1^ (4C), respectively. Moreover, when cycling back to low rate from high rate, the TaS_3_ NF electrode retains more reversible capacity (*ca.* 92%; 65th, 169 mA h g^–1^ out of fifth, 186 mA h g^–1^) than the bulk electrode (*ca.* 78%; 65th, 137 mA h g^–1^ out of fifth, 175 mA h g^–1^). The selected (dis)­charge curves at various current densities in the rate measurement are provided in Figure S13. As shown in Figure 3e, when evaluating the long-term cycling performance at high current density (after 10 initial cycles at 0.2C), the TaS_3_ NF electrode exhibits a capacity of 110.7 mA h g^–1^ after 1000 cycles at 2C, corresponding to 73.1% of the starting capacity, while the bulk TaS_3_ electrode only retains 67.3% (97.2 mA h g^–1^ out of 144.2 mA h g^–1^) after approximately half the equivalent number of cycles. The results again corroborate the benefits of employing the nanofibrous, flexible TaS_3_ film as binder-free electrode for improving cycling stability and capacity, especially at high rates when potential structural damage from exfoliation/detachment is more pronounced.

The morphology changes that the TaS_3_ NFs undergo on cycling were investigated using SEM. Figure 4 displays SEM images of TaS_3_ NF electrodes in the uncharged state, and after the first, second, and 50th charge/discharge cycles. The most notable evolution observed is a progressive exfoliation of the original fibers along the fiber length direction during the initial two (dis)­charge cycles, resulting in the formation of numerous thin saw-like nanoribbons with a thickness of less than 100 nm and lateral sizes ranging from 200 to 500 nm. Clear evidence of electrochemical exfoliation can be observed in Figure 4b-e, where several peeling regions are marked with yellow dashed circles. Moreover, the detailed peeling joints and a representative example showing substantial exfoliation of large fibers are provided in Figure S14. After the 50th charge, it can be observed that the lateral size of the fibers remains of the same order of magnitude to that after the first two cycles, indicating that exfoliation mainly occurs in the early discharge and charge processes (Figures 4f and S14f). Photographs of the TaS_3_ NF electrode after the 50th charge cycle highlight that the self-standing flexibility of the film was well preserved (Figure S15), underscoring the robustness of the TaS_3_ NF electrode and its suitability for potential integration into wearable energy storage devices. The mechanical resilience of the electrode ensures its ability to adapt to the dynamic and deformable environments required for wearable applications, where flexibility and stable performance are critical. The morphological change, associated with the exfoliation of TaS_3_ NFs, is believed to result from the volume change induced by the intercalation of Li^+^ cations. Similar behavior has been reported in other transition metal trichalcogenides, such as TiS_3_, HfS_3_, and ZrS_3_.^28,45^ A possible explanation is provided below. Similar to other trichalcogenides in the literature, TaS_3_ tends to form micro- to nanosized crystals with high aspect ratios due to its quasi-1D [TaS_6_]_*n*_ chain structure. These chains are assembled via weak interchain interactions, which makes cleavage along planes perpendicular to the chain direction (the *b*-axis) relatively easy. TiS_3_ has previously been shown to have low theoretical cleavage energies of 0.714, 0.716, and 0.815 J m^–2^ to separate the [TiS_6_]_*n*_ chains along the (101), (101̅), and (100) planes, respectively, as compared to the lowest cleavage energies of 0.320 J m^–2^ along the (001) planes for the exfoliation of pseudolayers, which contrast to the higher values of up to 2.706 J m^–2^ required to break the intrachain covalent bonds along the (010) planes. In another study, O̅nuki *et al.* directly observed that the width of a bulk ZrS_3_ single crystal along the *a*-axis expanded significantly after chemical lithiation using *n*-butyllithium solution, whereas the length along the *b*-axis remained nearly unchanged.^28^ These previous observations indicate that separation between chains in trichalcogenides is energetically more favorable than breaking the chains themselves. Therefore, it can be deduced that when additional stress is induced in the structure of TaS_3_ by the intercalation and deintercalation of Li^+^ ions, the weak interchain interactions allow the [TaS_6_]_*n*_ chains to expand and separate easily. This process could lead to the exfoliation of pristine TaS_3_ fibers into considerably smaller ribbon crystals while maintaining high aspect ratios. The exfoliation process causes little collapse or detachment of the nanofibers/ribbons during cycling, preserving the length and high aspect ratios of the nanoribbons and maintaining the robust interconnected fibrous structure. This phenomenon helps rationalize the relatively stable cycling performance (i.e., high capacity retention after extended cycles and under high rates) of the NFs in comparison to bulk TaS_3_.

**Figure 4:**
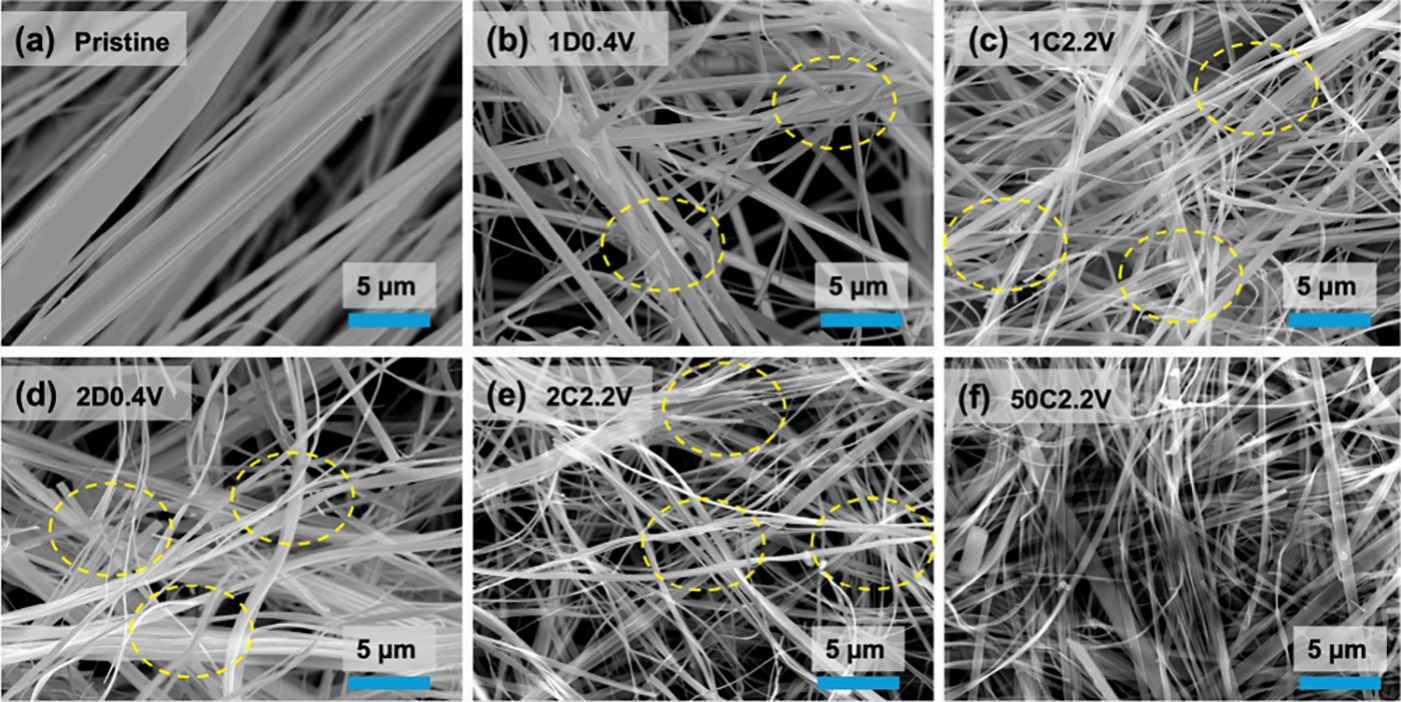
SEM images of the TaS_3_ NF electrode materials in the (a) uncharged, pristine state, and after the first (b) discharge (1D0.4 V) and (c) charge (1C2.2 V), (d) the second discharge (2D0.4 V) and (e) charge (2C2.2 V), and (f) the 50th charge (50C2.2 V) cycles. The yellow dashed circles highlight the regions where exfoliations occur.

XPS was employed to investigate the presence of Li and Mg, as well as the chemical states of Ta and S during the first two (dis)­charge cycles. Figure 5a displays the high-resolution spectra in the Li 1s and Mg 2p regions. It is evident that in the first and second discharged (1D0.4 V and 2D0.4 V) states, the Li 1s and Mg 2p peaks are present. Although the intensity and resolution in the Li 1s and Mg 2s region are relatively low, it is evident that upon charging, the intensity of the Li 1s peak noticeably decreases, while the Mg 2p peak changes very little. EDS results (Figure S16 and Table S5) of the cycled electrodes further reveal a very limited Mg content (equivalent to *ca.* 15 mA h g^–1^), along with subtle yet observable variations between the first and second cycles. This suggests that Li^+^ cations serves as the primary charge carriers in TaS_3_ NF, while Mg^2+^ contributes only marginally through partially reversible storage. This reversible component of Mg storage is likely associated with pseudo capacitive behavior enabled by electrochemically induced fiber thinning and increased surface accessibility after the first cycle. The fitted Ta 4f regions of the TaS_3_ electrode at different states are presented in Figure 5b. It can be observed that (the first and second) discharge of the TaS_3_ NF electrodes lead to the emergence of new peaks/intensity at lower binding energies of *ca*. 22.7 and 24.6 eV, attributable to the 4f_7/2_ and 4f_5/2_ transitions of Ta^3+^,^46^ respectively. As minimal changes are observed in the peak intensities of Ta^4+^ species within the S_2_^2–^-containing chains - and given that the reduction of these Ta^4+^ species is not expected above 0.4 V, similar to TiS_3_ ^28^ - it is inferred that Ta^5+^ species in the S_2_^2–^-free chains undergo reduction to Ta^3+^ during discharge cycles. Upon charging, these doublet peaks shift back to higher binding energies of *ca.* 24.2 and 26.2 eV that represent the Ta^5+^ state of TaS_3_, suggesting reversible oxidation of Ta upon extracting Li^+^ ions. Peaks for Ta^4+^ (*ca.* 25.0 and 27.0 eV) and Ta^5+^ (*ca.* 25.9 and 27.9 eV) from respective oxides are also present in the spectra, possibly due to the oxidation of the electrode materials during electrode washing, handling, and the transfer to the instrument. In the S 2p region of the high-resolution spectra (Figure 5c), the doublet peaks for S_2_^2–^ completely disappear after the first discharge, suggesting the reduction of S­(-I) to S­(-II). Upon charging, S in the form of S_2_^2–^ partially recovers. The second discharge–charge cycle follows a similar pattern to the first cycle. Notably, the characteristic binding energies of S^2–^ and S_2_^2–^ species shift to slightly lower and higher values after discharge and charge, respectively. This phenomenon was also observed in other disulfide-containing compounds (e.g., amorphous TiS_4_) during lithiation or delithiation, and was attributed to the increased (lower S 2p binding energy) or decreased (higher S 2p binding energy) ionic character of S^2–^ and S_2_^2–^ species coordinated with transition metal and Li ions in the electrode compound.^47^ XAS S K-edge spectra of the TaS_3_ NF electrodes at various discharge and charge states were also measured for the corroboration of the S_2_^2–^/S^2–^ anionic redox reaction. As shown in Figure 5d, the XAS spectrum of the pristine TaS_3_ NF electrode displays a pre-edge peak at *ca.* 2456.6 eV (marked as “peak *a*”), which arises from transitions to unoccupied S 3p states hybridized with Ta 5d bands.^48^ Additionally, the other peak positioned at *ca.* 2464.3 eV (“peak *b*”) can be assigned to transitions to higher energy states, such as S 3p states hybridized with Ta 6s or 6p bands, corresponding to the complete ejection of the core electron into the continuum.^48^ After the first discharge, peak *a* shifts to lower photon energy and splits into two weak, overlapping peaks at *ca*. 2453.4 and 2455.2 eV, while peak *b* shifts to a lower energy position of 2460.5 eV. These peak shifts indicate an increase in the electron density around sulfur and a decrease in the effective nuclear charge, providing strong evidence for the reduction of S­(-I) to S­(-II) anions. The splitting of peak *a* is likely caused by Li^+^ intercalation, which alters the local electrostatic interactions of sulfur anions with tantalum d orbitals.^48^ This may involve a coordination change in tantalum, transitioning from trigonal prismatic to octahedral symmetry.^48^ Upon the first charge, peaks *a* and *b* return to their higher energy positions, with the splitting of peak *a* disappearing. This observation indicates the reversible oxidation of S­(-II) anions back to S­(-I). The behavior in subsequent cycles closely resembles that of the first cycle. These XPS and XAS results unequivocally confirm the electrochemical activity of sulfur anions and tantalum cations in the TaS_3_ NF electrode. Together, these findings support a dual redox mechanism in which both sulfur and tantalum contribute to charge storage with complementary roles. Analogous reaction mechanisms can be found in VS_4_ in MIBs,^25^ Li_2_TiSe_3_ in LIBs,^49^ and Na_2_FeS_2_ in SIBs.^50^

**Figure 5:**
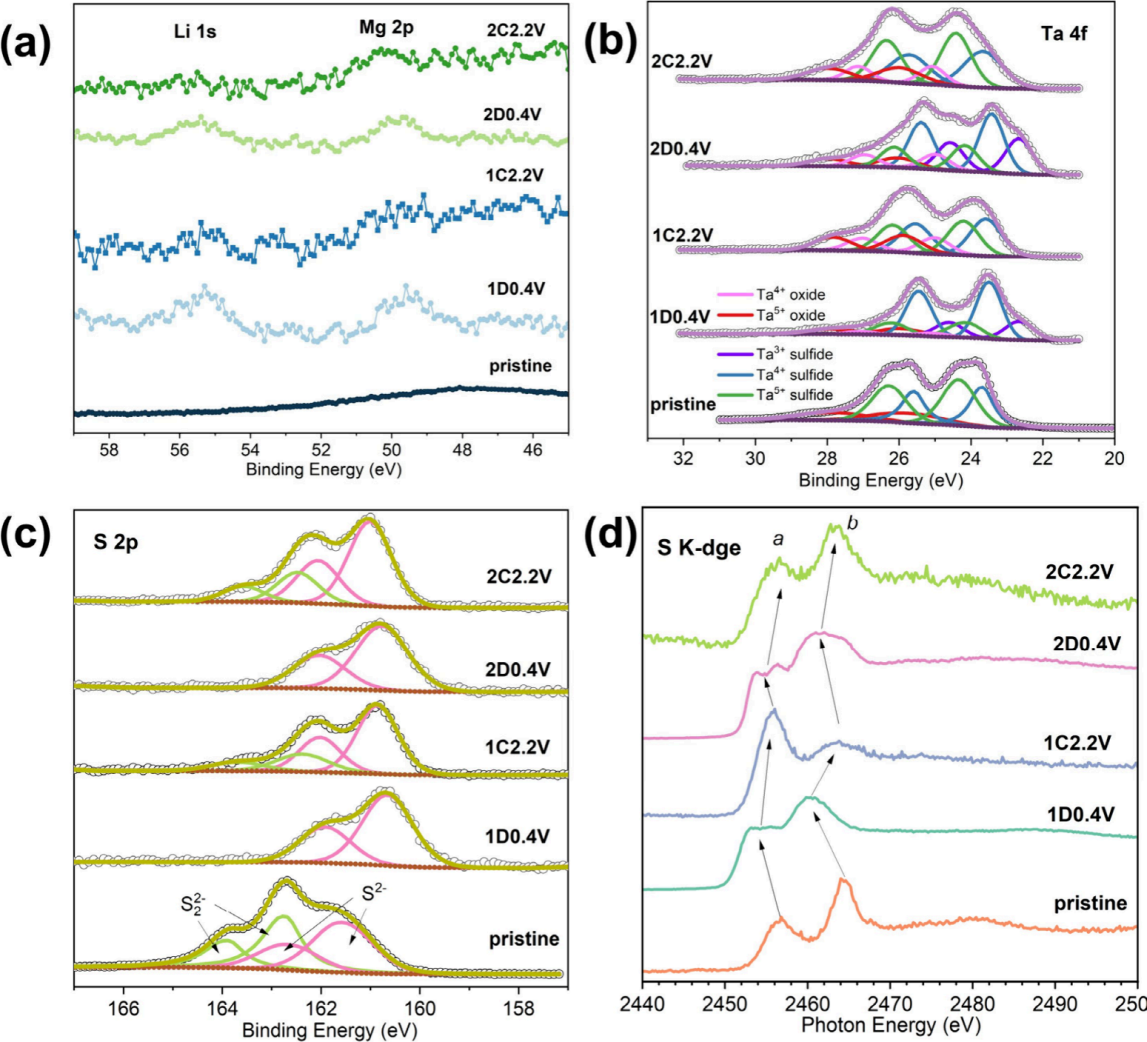
High-resolution XPS spectra of (a) Li 1s and Mg 2p, (b) Ta 4f, and (c) S 2p regions, and (d) the S K-edge XAS spectra of the TaS_3_ NF electrode at different (dis)­charge states.

To gain insight into the structural evolution during electrochemical Li^+^ (and trace amount of Mg^2+^) cation insertion/deinsertion, *in operando* PXRD experiments were conducted, and the data were processed and plotted as contour graphs. Figure 6a displays the *in operando* PXRD patterns of the TaS_3_ NF electrode during the initial three (dis)­charge cycles. During the first discharge, all the peaks for TaS_3_ NFs progressively fade away and ultimately disappear by the end of discharge at 0.4 V. Subsequently, a new set of peaks gradually emerge at approximately 11.0°, 12.6°, 14.0°, 16.0°, 17.3°, 26.6°, 28.7°, 34.9°, 48.3°, 50.4°, and 60.3° (all 2*θ*), indicating the formation of (a) new lithium-intercalated phase(s). As the 1D fiber/ribbon-like morphologies of the discharged electrode material are well maintained, it could be assumed that the presence of these new peaks may correspond to the arrangement of 1D [TaS_6_]_*n*_ chains with the incorporation of Li^+^ (and trace Mg^2+^) ions. The transmission mode *ex situ* PXRD patterns of the first discharged electrode and the mechanically synthesized cubic Li_2_TaS_3_ are shown in Figure S17. It is observed that the pattern of the TaS_3_ NF electrode shows an obvious mismatch with those of monoclinic (calculated based on monoclinic Li_2_NbS_3_) and as-made cubic Li_2_TaS_3_, indicating that the intercalation of Li^+^ (and trace amount of Mg^2+^) does not result in the formation of these phases. Therefore, these new peaks are attributed to a phase (phase M) of composition Li_*d*_Mg_*f*_TaS_3_ (*d* < 2; *f* ≈ 0) based on the evolution of the peaks in the subsequent cycles. During the first charging process, the peaks for phase M first shift to slightly higher angles and then disappear by the maximum cutoff voltage of 2.2 V, whereas broad scattering regions (marked by blue arrows; Figure 6b and Figure S18) subsequently emerge at the end of the charging process. These features indicate the formation of an amorphous TaS_3_-like phase (“*pseudo*-TaS_3_”) after deintercalation of most of the Li^+^ (and trace amounts of Mg^2+^) ions. However, there are still *ca.* 10.3 at. % of Li^+^ (and a trace amount of Mg^2+^) ions trapped in the *pseudo*-TaS_3_, possibly making the details of the local structure subtly different compared to that of the pristine TaS_3_ NF electrode. These observations suggest that the extraction of Li^+^ (and trace amounts of Mg^2+^) is only partially reversible. In the subsequent two cycles, phase M remains present with the (de)­intercalation of Li^+^ (and trace Mg^2+^) ions, although its diffraction peak at *ca.* 12.6° tends to shift to a higher 2*θ* position. However, the scattering for *pseudo*-TaS_3_ gradually fades and eventually disappear upon extended cycling. Indeed, when the *in operando* PXRD patterns were collected from the 35th charged electrode (Figure S19a), *pseudo*-TaS_3_ was no longer detectable. Meanwhile, the presence of the M phase strengthened, showing a typical zigzag-style continuous variation of PXRD patterns possibly indicating a solid solution reaction within Li_*d*_Mg_*f*_TaS_3_ (*d* < 2; *f* ≈ 0) upon intercalation (lattice expansion) and deintercalation (lattice contraction) (Figure S19b).^51^ The Raman spectra of the TaS_3_ NF electrodes during the initial 2 cycles were collected and are compared in Figure 6c. In the spectrum of the first discharged (1D) electrode, signals for pristine TaS_3_ completely disappear and several new broad peaks arise, indicating a highly disordered structure in phase M. Most notably, the broad peak at approximately 425 cm^–1^ is attributed to Ta–S stretching mixed with Li–S bond stretching, similar to the behavior reported for Li_2_TiS_3_,^52^ while the signals at approximately 283 cm^–1^, 185 cm^–1^, and 147 cm^–1^ could be assigned to the Li–S vibration mode in the lithiated electrode (Li_2_TaS_3_). After charging, the vibration modes for Li_2_TaS_3_ are replaced by another set of broad peaks, which can be ascribed to highly disordered and poorly crystallized *pseudo*-TaS_3_. The reappearance of the stretching mode of disulfide bridges at approximately 490 cm^–1^ demonstrates the reversible S_2_^2–^/S^2–^ anionic redox in the TaS_3_ NF electrode. The second cycle exhibits similar behavior to the first cycle, except for a slight red shift of peaks, possibly due to structural adjustment resulting from the trapping of some Li^+^ cations in the lattice host. Although the structures of the TaS_3_ NF electrodes at 1D0.4 V and 2D0.4 V states differ significantly from the as-synthesized cubic Li_2_TaS_3_, their Raman spectra appear to be quite similar. Several factors could contribute to this: (a) the Raman bands are predominantly influenced by Li–S bond vibrations,^53^ which could overshadow other structural features related to Ta–S bonding and lead to spectral similarities despite the distinct structural phases; (b) the low resolution of the Raman spectra for both the highly disordered discharged TaS_3_ NF electrodes and cubic Li_2_TaS_3_ complicates the precise descrimination between subtle Ta–S stretching modes, potentially masking structural differences.

**Figure 6:**
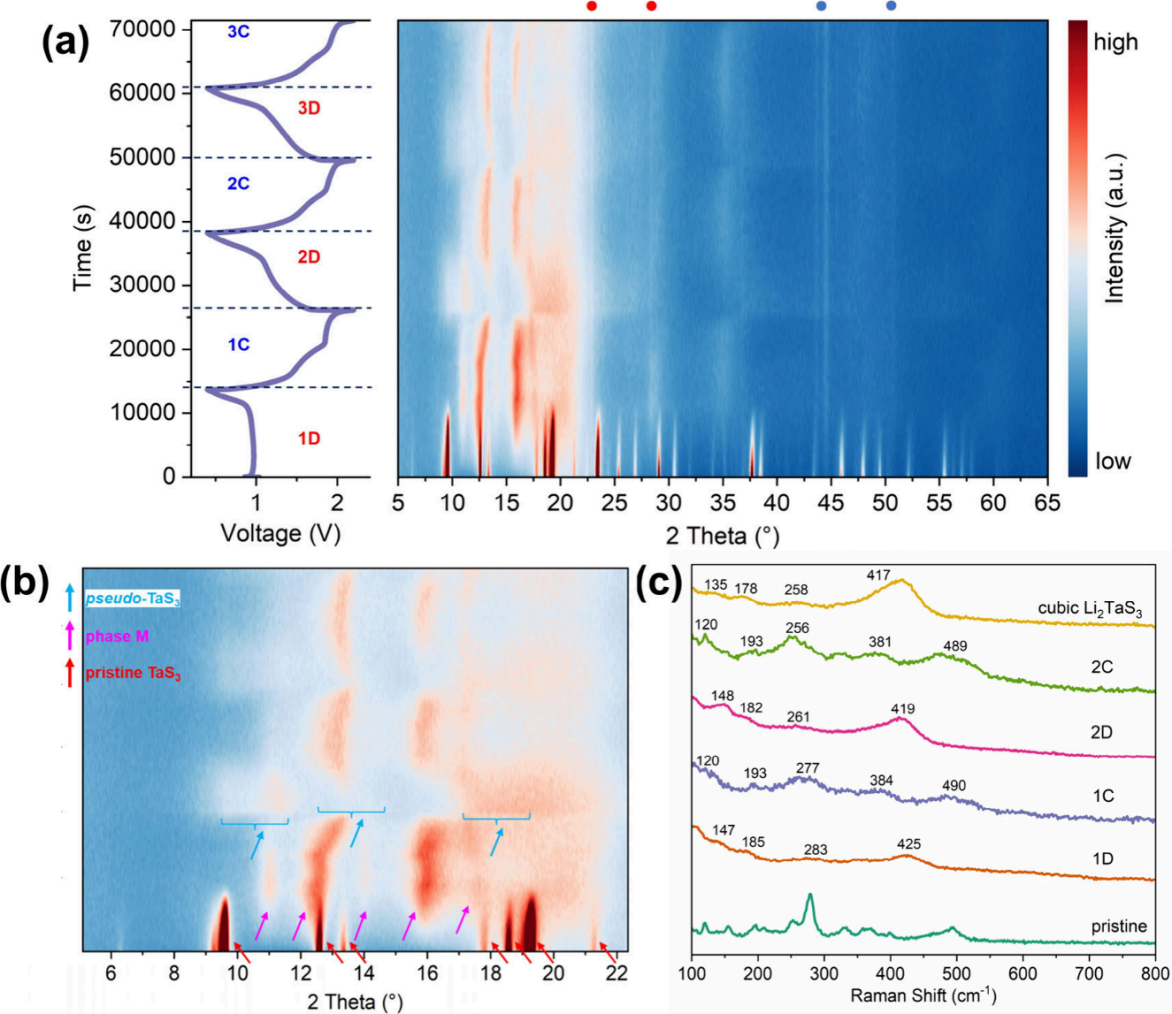
(a) Initial three (dis)­charge curves (left) of the TaS_3_ NF electrode obtained at a current density of 50 mA g^–1^ and their contour plots (right) of the corresponding *in operando* PXRD patterns. Note that the red marks indicate the presence of an inactive unknown minor impurity in the electrode material *in operando* cell, and blue marks indicate the diffraction of stainless steel from the cell casing. (b) Zoomed *in operando* PXRD plot taken from (a), in which red, pink, and blue arrows represent the peaks for pristine TaS_3_ NF, phase M, and *pseudo*-TaS_3_, respectively. (c) Raman spectra of the uncycled, the first discharge (1D0.4V), first charge (1C2.2 V), second discharge (2D0.4 V), and second charge (2C2.2 V) states of the TaS_3_ NF electrode.

Subsequently, a series of electrochemical techniques were employed to investigate the charge storage behavior and kinetics of the TaS_3_ NF electrode. To explore the charge storage mechanism electrochemically, CV experiments were conducted, and the resulting CV curves were collected at various scan rates of 0.1 mV s^–1^, 0.2 mV s^–1^, 0.3 mV s^–1^, 0.4 mV s^–1^, and 0.5 mV s^–1^. The experimental data presented in Figure 7a were analyzed using Equations S1–2. Notably, the *b*  parameter typically ranges from 0.5 to 1.0. A *b* value of 1.0 (*I* = *av*) indicates pseudo capacitance-controlled behavior, while a *b* value of 0.5 (*I* = *av* ^1/2^) signifies a diffusion-controlled process. The cathodic/anodic peak currents were collected and plotted as the log values against log *v* accordingly (Figure 7b). The linear fits to these data produced *b* values of 0.863 ± 0.003 and 0.715 ± 0.009 for the two dominant cathodic/anodic peaks, indicating a mixed charge storage behavior. Pseudo capacitance-controlled behavior represents rapid ion adsorption and/or intercalation on the (near) surface of the nanofibers, which favors cycling performance at high current densities, while diffusion-controlled behavior represents the intercalation of Li^+^ cations into the bulk crystal of the TaS_3_ material.^54^ Similar blended charge storage mechanisms to that observed have been reported in the literature, for example, in TiNb_2_O_7_ (*b* = 0.816) and VS_2_ nanosheets (*b* = 0.790/0.883) in MLIBs,^55,56^ and in TiO_2_ nanocrystals (*b* = 0.787 and 0.787) in MIBs.^57^

**Figure 7:**
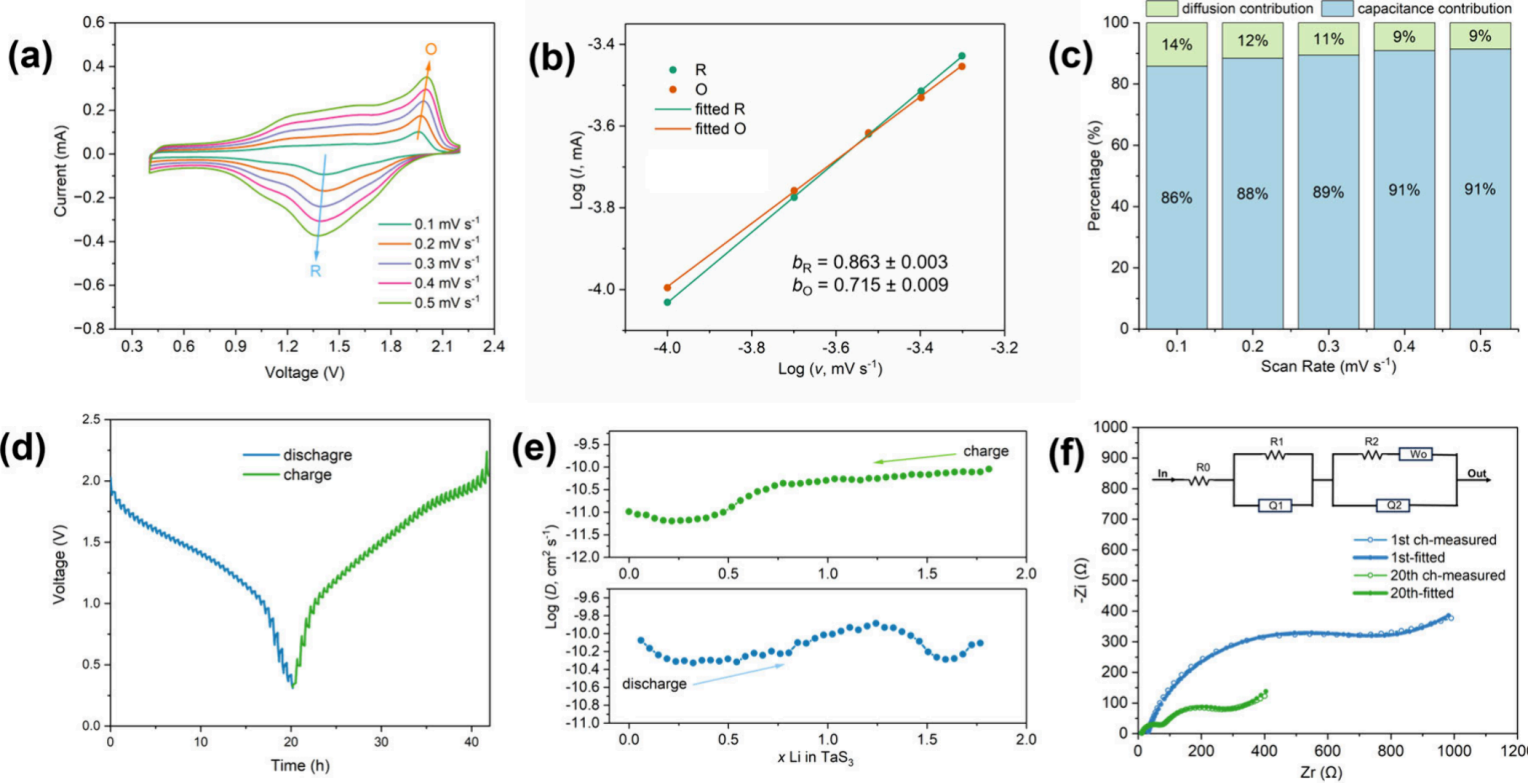
Charge storage mechanism, Li^+^ ion diffusion kinetics, and interfacial resistance properties of the TaS_3_ NF electrode: (a) CV curves obtained at different scan rates of 0.1 mV s^–1^ (green), 0.2 mV s^–1^ (orange), 0.3 mV s^–1^ (violet), 0.4 mV s^–1^ (pink), and 0.5 mV s^–1^ (light green), respectively. (b) Plots of the measured and fitted log reductive (green) and log oxidative (orange) peak currents against the log of scan rates *v*, in which the solid lines represent the linear fits (adjusted R-squared values, R_R_^2^ = 0.9999, R_O_^2^ = 0.9995). (c) Histogram of the separate capacitance and diffusion contributions to the charge storage at various scan rates. (d) Discharge and charge GITT curves at a current density of 25 mA g^–1^. (e) Plots of discharge and charge diffusivities against Li^+^ cation level applying the data derived from the GITT curves. (f) Measured (thin lines with open circles) and fitted (thick lines) EIS spectra for the (−)­Mg|LiAPC|TaS_3_ NFs­(+) cells after the first charge and the 20th charge cycles. The equivalent circuit is shown as an inset in the graph.

The proportion of pseudo capacitance- and diffusion-controlled charge storage was further determined using Equation S3. By linearly fitting data for *i*/*v* ^1/2^ against 1/*v* ^1/2^ (obtained from Figure 7a), both constants were derived and used to deduce the relative proportions of the pseudo capacitance- and diffusion-controlled charge storage contributions. At scan rates of 0.1 mV s^–1^, 0.2 mV s^–1^, 0.3 mV s^–1^, 0.4 mV s^–1^, and 0.5 mV s^–1^, the percentages of pseudo capacitance in TaS_3_ NF are 86%, 88%, 89%, 91%, and 91%, respectively (Figure 7c). The substantial proportion of pseudo capacitative charge storage suggests rapid charge transfer and ion transport occurring on the (near) surface during (dis)­charge processes, particularly when the fibers were appreciably exfoliated and became increasingly thin (as observed from the electrode morphology in extended cycles). The presence of considerable pseudo capacitance undoubtedly enhances the rate performance of the TaS_3_ NF electrode, as evidenced by the voltage–time profiles at high current densities (discharged and charged within *ca.* 17 and 7 min at 500 and 1000 mA g^–1^, respectively; Figure S20)

To further investigate and analyze the kinetics of the Li^+^ ion diffusion (neglecting the trace amount of Mg^2+^) in the structure of the TaS_3_ NF electrode material, GITT experiments were conducted, with detailed procedures outlined in the Supporting Information. Subsequently, Li^+^ cation diffusion coefficients (*D*) were calculated based on Equation S4 and the as-measured GITT curves (Figure 7d). These values, as a function of *x* (Li_*x*_TaS_3_) at various discharge and charge states, were plotted, as depicted in Figure 7e. During the discharge and charge processes, diffusion coefficients range from 4.7 × 10^–11^ cm^2^ s^–1^ to 1.3 × 10^–10^ cm^2^ s^–1^ and from 6.4 × 10^–12^ cm^2^ s^–1^ to 9.1 × 10^–11^ cm^2^ s^–1^, respectively. Notably, two broad peaks reveal minimum diffusivities of 4.7 × 10^–11^ cm^2^ s^–1^ and 5.2 × 10^–11^ cm^2^ s^–1^ at discharge states approximating to “Li_0.32_TaS_3_” and “Li_1.59_TaS_3_”, respectively, suggesting a two-stage Li^+^ cation intercalation diffusion in the host lattice. In the charging process, as Li^+^ cations are extracted from the lattice, the diffusivity steadily decreases to a point of 4.3 × 10^–11^ cm^2^ s^–1^ after it passes a minimum diffusivity of 6.4 × 10^–12^ cm^2^ s^–1^. The slight disparity between the discharge and charge diffusivity profiles may stem from the slightly different Li^+^ ion diffusion pathways during insertion/extraction, influenced by the adjustment of the extended and local structures upon incorporating/releasing Li^+^ ions, a phenomenon also noted in other cases.^15,58^ In short, the obtained diffusion coefficients (average *D*_discharge_ of *ca.* 7.1 × 10^–11^ cm^2^ s^–1^) are comparable to or higher than other chalcogenides, such as VS_4_, VS_2_, TiS_2_, and MoS_2_, reported as cathode materials for MLIBs in the literature,^15,21,58–60^ indicating good ion diffusion kinetics in the TaS_3_ NF electrode.

EIS was conducted to elucidate the charge transfer resistance as well as interfacial properties and ion diffusion processes in the TaS_3_ NF electrode. Figure 7f and Figure S22 show the obtained Nyquist plots and corresponding equivalent circuits (fitted using Aftermath software^33^) of three selected Mg|LiAPC|TaS_3_ NFs cells (before cycling, after the first charge, and after the 20th charge at a current density of 50 mA g^–1^). The modified Randles circuit model consists of three resistors (R_0_, R_1_, and R_2_), two CPEs (CPE_1_ and CPE_2_), and a Warburg impedance (W_0_). In the Nyquist plots, the starting point (R_0_) of the curve from *Z*_*r*_-axis represents the internal resistance caused by electrolyte, current collector, *etc*. The high-frequency region is modeled as a combination of resistance and capacitive elements (R_1_ + CPE_1_ in parallel), corresponding to the solid-electrolyte-interphase (SEI) film. The remaining section of the model ((CPE_2_ + W_0_) and Q_2_ in parallel) accounts for a blend of charge transfer and diffusion-mediated behavior in the electrodes.^61^ Detailed fitting results (Table S6) reveal significant changes in interphase resistance (R_1_) and charge transfer/diffusion behavior (R_2_ and W_0_). Notably, R_1_ decreases substantially from *ca.* 3296 Ω to *ca.* 21 Ω after the first charge, indicating a modification process involving the removal of passivation or oxidized layers on the TaS_3_ NF electrode and the Mg anode. After 20 cycles, the R_1_ element increases slightly to *ca.* 55 Ω, likely due to the establishment of a thickened electrolyte-electrode surface following adsorption after the interfacial modification process and exfoliation observed in the SEM results (exposing new nucleation surfaces). Regarding charge transfer/diffusion behavior, R_2_ decreases significantly to *ca.* 742 Ω after the first charge from *ca.* 7999 Ω in the uncycled electrode and drops further to 230 Ω after 20 cycles. Concurrently, the Warburg impedance W_0_ decreases continuously to *ca.* 146 Ω s^–1/2^ after 20 cycles from the initial large value (*ca.* 794 Ω s^–1/2^). These results suggest that charge transfer and Li^+^ cation diffusion in the TaS_3_ NF electrode improve gradually upon initiating the cycling measurement, owing to a so-called “activation process” involving the electrochemical cleaning of passivated or oxidized surface layers, as frequently observed in similar systems.^25,56^

Based on the above findings, we further provide the following perspectives on the reliability of the current electrode architecture and future material design strategies. First, the self-standing fibrous architecture plays a pivotal role in the cycling stability of TaS_3_: it mitigates exfoliation-induced active material loss while preserving structural integrity, and simultaneously takes advantage of *in situ* nanosizing caused by exfoliation, which leads to shortened ion migration pathways and enhanced pseudo capacitive behavior. Second, such nondestructive exfoliation within the fibrous film framework does not progress indefinitely; rather, it is largely completed after the second cycle. This precludes the possibility that newly exposed active sites are the primary contributor to capacity retention during extended cycling. Third, although complex phase transitions occur during the initial cycles - driving exfoliation - the structural evolution becomes more homogeneous and stabilizes into a solid-solution-like behavior over prolonged cycling (Figure S18), thereby reducing further volume changes and exfoliation, as evidenced by the stable morphology after 50 cycles (Figure 4f). This structural stabilization also underpins the long-term cycling performance observed in our system.

However, such exfoliation-associated volume changes may present challenges in practical full-cell configurations. Moreover, the resulting *in situ* nanosizing can increase side reactions with the electrolyte, leading to a thicker SEI (Figure 7f). Future improvement may focus on a pretreatment strategy involving chemical intercalation and subsequent oxidation to produce a pre-exfoliated fiber film, which could alleviate volume changes and suppress excessive SEI formation during operation.

## 4 Conclusions

In this work, self-standing flexible TaS_3_ NF films have been synthesized using the PVT method and introduced as cathode materials in MLIBs for the first time. This electrode, devoid of carbon, binder, and supporting matrix, circumvents capacity/energy density loss resulting from inactive battery components. Moreover, the uniform structure of the film electrodes, comprised of numerous cross-linked nanofibers, ensures reliable electrical conductivity and stable charge transfer and ion transport. Consequently, the TaS_3_ NF electrode exhibits promising electrochemical performance, including high rate performance, very stable cycling, and superior capacity retention (91.6%), compared to powdered TaS_3_ (56.1%). Mechanistic studies shed light on the structure, morphology, and electrochemistry underlying the performance of the TaS_3_ NF electrode. Analysis of postcycle morphological changes suggests that maintaining the integrity of microstructure during exfoliation is pivotal in bringing about the excellent performance, contrasting to the powdered trisulfides, which suffer from the detachment of active materials. TaS_3_ NF electrodes undergo a partially reversible phase transition to form a Li_2_TaS_3_-like phase, M, in the initial cycles. This phase transition behavior gradually diminishes, with structural expansion and contraction occurring solely within phase M during extended cycling. The ion storage capacity arises from a dual redox mechanism involving the S_2_^2–^/S^2–^ anionic redox and the cationic couple Ta^5+^/Ta^3+^, together with a large proportion of pseudo capacitance originating from the nanosizing effect of exfoliated fibers. Additionally, the demonstrated retention of flexibility, even after extended cycling, highlights the electrode’s potential for integration into wearable energy storage devices. Although related tantalum materials such as TaS_2_, Ta_2_O_5_, and metallic Ta have demonstrated good biocompatibility in prior studies,^62–64^ the biosafety of TaS_3_ specifically has yet to be systematically evaluated. Future studies involving *in vitro* and *in vivo* biological assessments are warranted to determine its suitability for such applications. This study provides the groundwork for the development of future high-performance rechargeable batteries through the design of self-standing, flexible, carbon- and binder-free electrodes, which could enable applications in next-generation flexible and wearable electronics.

## Supplementary Material



## Notes

Digital photos of synthesis and products, PXRD patterns, Rietveld refinement results, SEM images, EDS spectra, TG-DSC curves, CV curves, (dis)­charge curves, and EIS spectrum (PDF)
