# Supplementary material for: Self-Supporting Quasi-1D TaS3 Nanofiber Films with Dual Cationic/Anionic Redox for High-Performance Mg–Li Hybrid Ion Batteries
Source: ACS Appl Mater Interfaces. 2025 Jul 23;17(31):44513–27. doi: 10.1021/acsami.5c09460 (PMC12332840; doi:10.1021/acsami.5c09460)
Supplement: Supplementary file 1 [file am5c09460_si_001.pdf]

Supporting Information

Self-Supporting Quasi-1D TaS_3 Nanofiber Films with Dual Cationic/Anionic Redox for High-Performance Mg-Li Hybrid Ion Batteries

Pengcheng Jing,[†] Atsushi Inoishi,[‡] Eiichi Kobayashi,[§] Chengcheng Zhao,[†] Peng Ren,[⊥] Isaac Abrahams,[⊥] Duncan H. Gregory*[†]

[†]WestCHEM, School of Chemistry, University of Glasgow, Joseph Black Building, Glasgow G12 8QQ, U.K.

[‡]Institute for Materials Chemistry and Engineering, Kyushu University, Kasuga-koen 6-1, Kasuga, Fukuoka 816-8580, Japan

[§]Kyushu Synchrotron Light Research Center, 8-7 Yayoigaoka, Tosu, Saga 841-0005, Japan

[⊥]Department of Chemistry, Queen Mary University of London, Mile End Road, London E1 4NS, U.K.

*E-mail: Duncan.Gregory@glasgow.ac.uk. Tel: +44-141-330-8128.

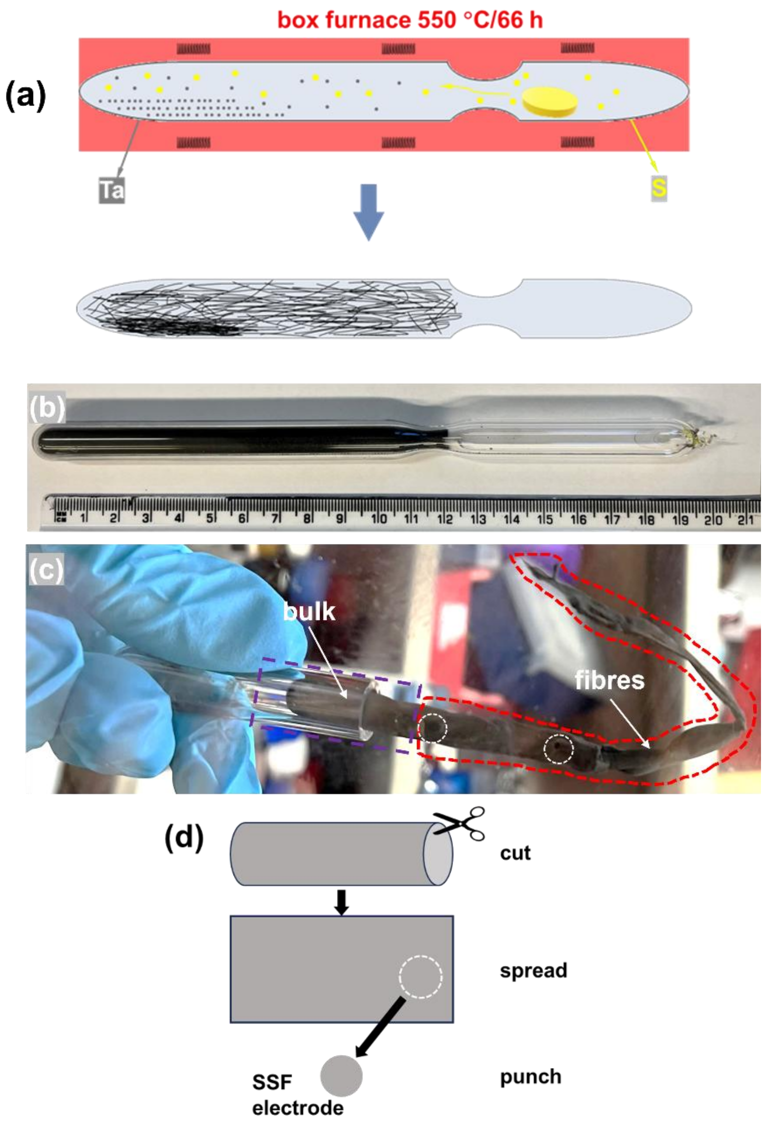

Figure S 1. (a) Schematic illustration for the synthesis of TaS_3 NFs. Photographs of (b) the quartz tube after heating and cooling to RT, and (c) a close-up showing the fibres (circled in red dashed lines; holes of the "leg" marked by white dashed circles) and bulk condensed deposit (circled in purple dashed lines). (d) Schematic illustration of the processes for forming an SSF TaS_3 NF electrode from fibre aggregates.

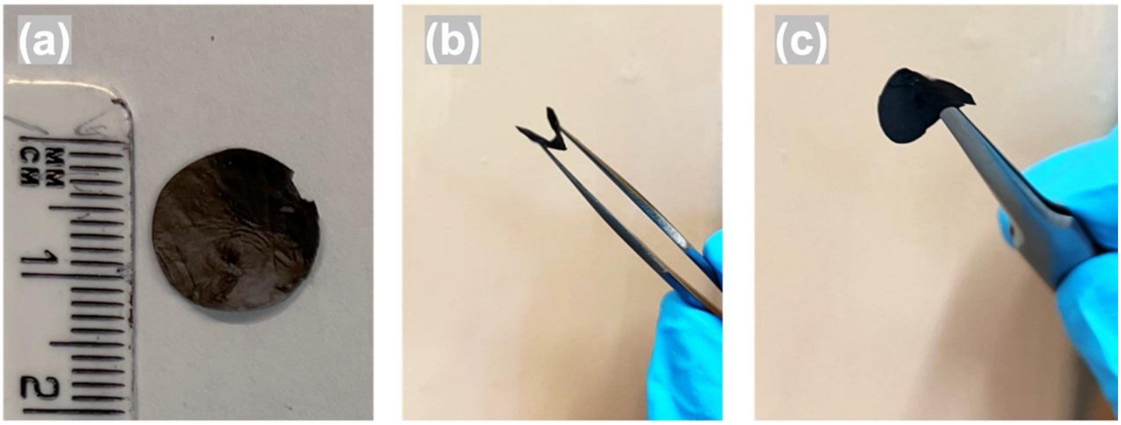

Figure S 2. (a) Self-standing TaS_3 nanofibre (NF) film after being collected from the tube and cut into disc with a diameter of 12 mm, and TaS_3 NF film (b) being folded to near 180 ° and (c) after recovery without fracturing.

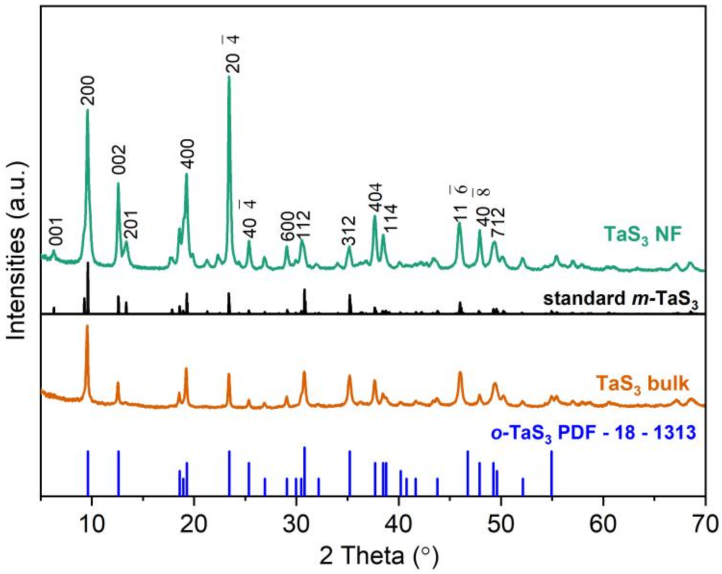

Figure S 3. Flat plate PXRD patterns of the as-synthesised TaS_3 NFs and bulk TaS_3, along with the standard *m*-TaS_3 pattern and PDF - 18 - 1313 for *o*-TaS_3.

Table S 1. Crystallographic data obtained from Rietveld refinement of TaS_3 NF as compared to the published data.[1]

Chemical Formula	m_1 phase in TaS_3 NF	Published m_1 phase
Crystal System	Monoclinic	Monoclinic
Space Group	$C\ 2/m$	$C\ 2/m$
Lattice Parameter	a = 19.9127(9) Å,<br>b = 3.3382(1) Å,<br>c = 15.1685(3) Å,<br>β = 112.463(5)°	a = 19.914(5) Å,<br>b = 3.337(3) Å,<br>c = 15.167(5) Å,<br>β = 112.4(4)°
No. of Points	7229	-
N – P + C	7193	-
Formula Weight / g mol^{-1}	276.9	276.9
Calculated Density / g cm^{-3}	5.93	5.92
Volume / Å^3	931.78(5)	934.05
R_{wp}	7.94%	-
R_p	5.47%	-
χ^2	4.62	-

Table S 2. Crystallographic data obtained from Rietveld refinement of m_2 phase TaS_3 NF as compared to the published data.[2]

Chemical Formula	m_2 phase in TaS_3 NF	Published m_2 phase
Crystal System	Monoclinic	Monoclinic
Space Group	$P\ 2_1/m$	$P\ 2_1/m$
Lattice Parameters	a = 9.527(3) Å, b = 3.332(2) Å, c = 14.933(7) Å, β = 110.23(3)°	a = 9.515(2) Å, b = 3.3412(4) Å, c = 14.912(2) Å, β = 109.99(2)°
No. of Points	7229	-
N – P + C	7193	-
Formula Weight / g mol^{-1}	276.9	276.9
Calculated Density / g cm^{-3}	6.20	6.20
Volume / Å^3	444.8(3)	445.51

Table S 3. Atomic position, B_{iso}, and occupancy parameters of Ta and S atoms in the unit cells of m_1-phase (from refinement) and m_2-phase (from the literature[1]).

m_1 **Phase**	Atom	Label	*x*	*y*	*z*	B_{iso}	*Occ.*
	Ta	Ta1	0.3503(2)	0.0000	0.0533(7)	0.4	1
	Ta	Ta2	0.6512(2)	-1.0000	0.4503(7)	0.4	1
	Ta	Ta3	0.3265(1)	-0.5000	-0.2094(5)	0.7	1
	S	S1	0.2736(13)	-0.5000	-0.0701(14)	0.9	1
	S	S2	0.4497(9)	-0.5000	0.1145(26)	1.1	1
	S	S3	0.6183(12)	-0.5000	0.5480(16)	1.1	1
	S	S4	0.2096(7)	-1.0000	-0.2658(21)	0.6	1
	S	S5	0.3797(13)	-0.5000	0.1836(15)	1.2	1
	S	S6	0.3989(10)	0.0000	-0.0746(15)	0.8	1
	S	S7	0.7274(13)	-0.5000	0.4115(14)	0.6	1
	S	S8	0.5559(8)	-0.5000	0.4055(26)	1.0	1
	S	S9	0.4042(11)	-1.0000	-0.2669(14)	0.8	1

m_2 **Phase**	Atom	Label	*x*	*y*	*z*	B_{iso}	*Occ.*
	Ta	Ta3	-0.2974	0.25	-0.4590	0.6	1
	Ta	Ta1	-0.6414	-0.75	-0.1192	0.5	1
	Ta	Ta2	-0.306	-0.25	-0.2023	0.5	1
	S	S6	-0.5149	-0.75	-0.2466	0.6	1
	S	S9	-0.243	-0.25	-0.5696	0.6	1
	S	S4	-0.1667	-0.75	-0.0834	0.5	1
	S	S7	-0.0885	-0.25	-0.4311	0.8	1
	S	S3	-0.8021	-1.25	-0.2365	0.6	1
	S	S2	-0.8296	-1.25	-0.1025	0.7	1
	S	S1	-0.4352	-1.25	-0.0636	0.6	1

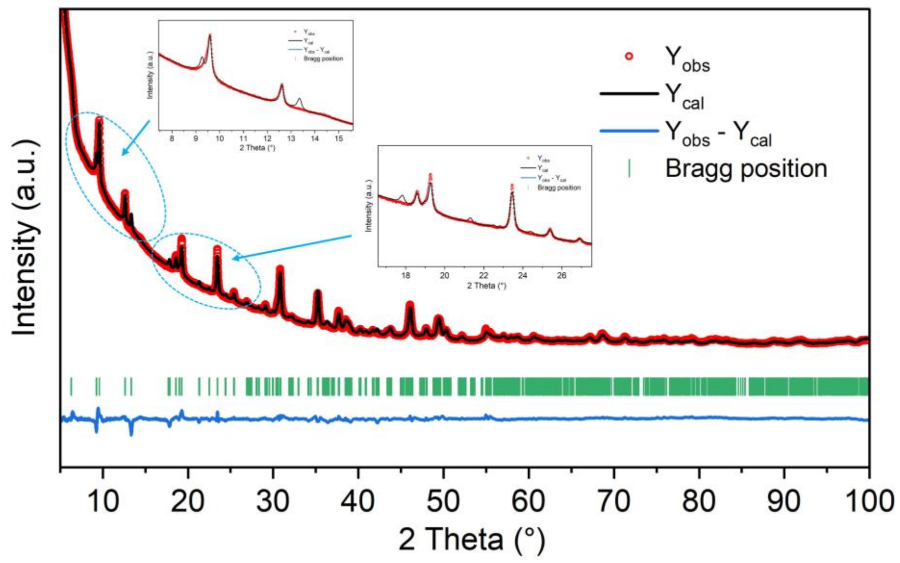

Figure S 4. Fitted PXRD pattern (transmission geometry, capillary mode) from Rietveld refinement for the bulk TaS_3 powder. Y_{obs} (red circles) and Y_{cal} (black line) represent the collected and refined intensities, respectively, while Y_{obs} - Y_{cal} (blue line) is the residual difference between Y_{obs} and Y_{cal} across the entire diffraction pattern. Green vertical lines represent Bragg positions.

Table S 4. Crystallographic data obtained from Rietveld refinement of the bulk TaS_3 powder.

Chemical Formula	Synthesised bulk TaS_3
Crystal System	Monoclinic
Space Group	*C* 2/*m*
Lattice Parameters	a = 19.9055(49) Å,<br>b = 3.3374(3) Å,<br>c = 15.1633(15) Å,<br>β = 112.363(31)°
No. of Points	7229
N – P + C	7132
Formula Weight / g mol^{-1}	276.9
Calculated Density / g cm^{-3}	5.928
Volume / Å^3	931.56(26)
R_{wp}	3.21%
R_p	1.96%
χ^2	0.37

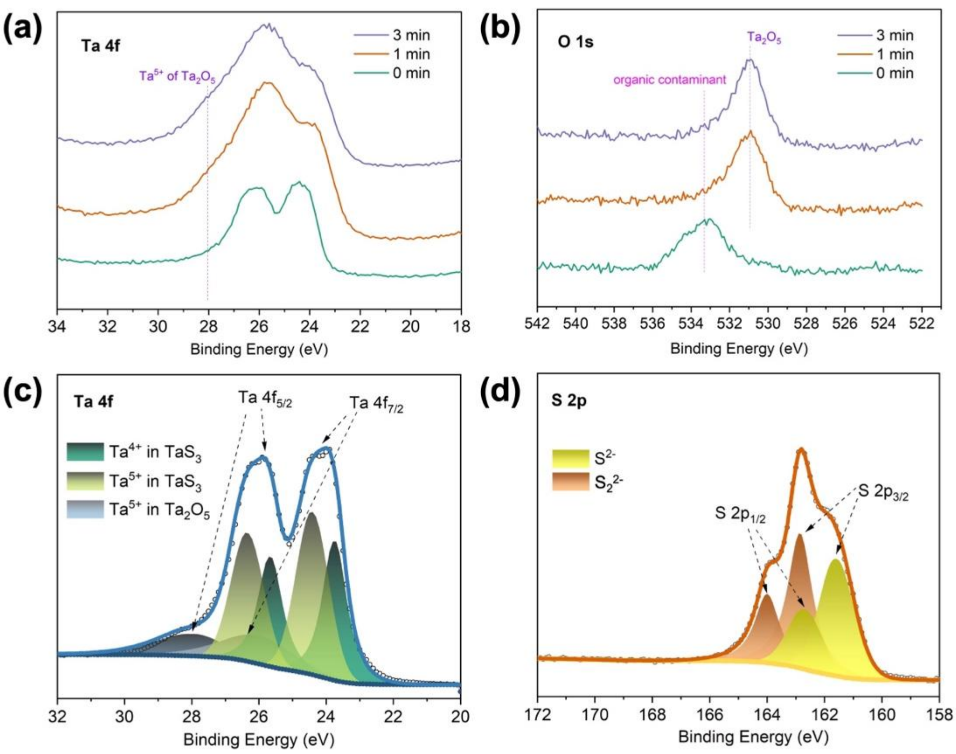

Figure S 5. XPS spectra of (a) Ta 4f and (b) O 1s regions of TaS_3 NF after Ar^+ etching for 0, 1, and 3 min, (c) Ta 4f and (d) S 2p regions in bulk TaS_3.

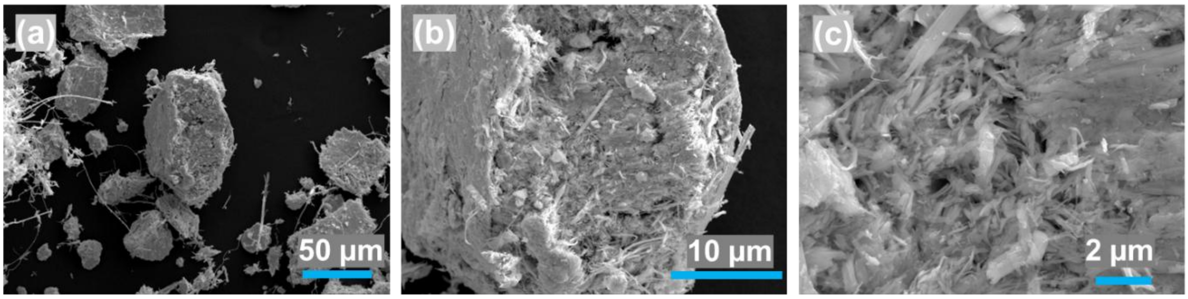

Figure S 6. (a)-(c) low- and high-magnification SEM images of the bulk TaS_3 powder.

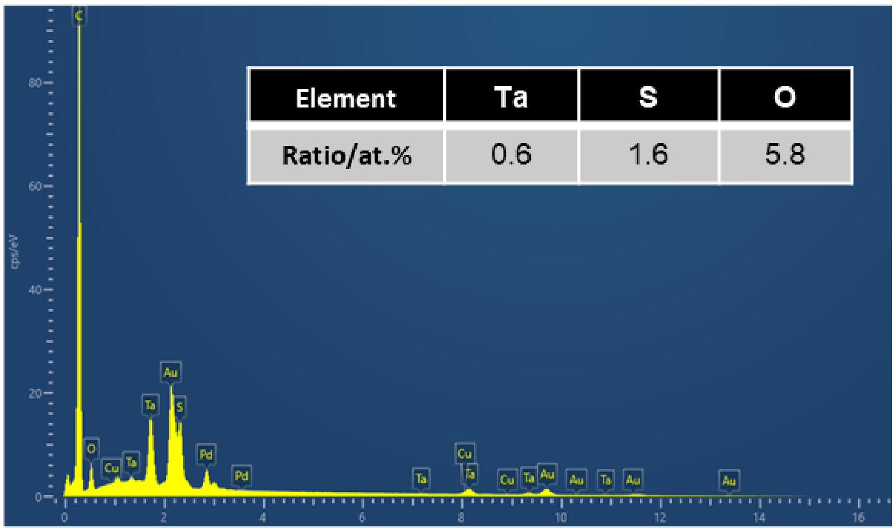

Figure S 7. EDS spectrum of the as-prepared TaS_3 NF film.

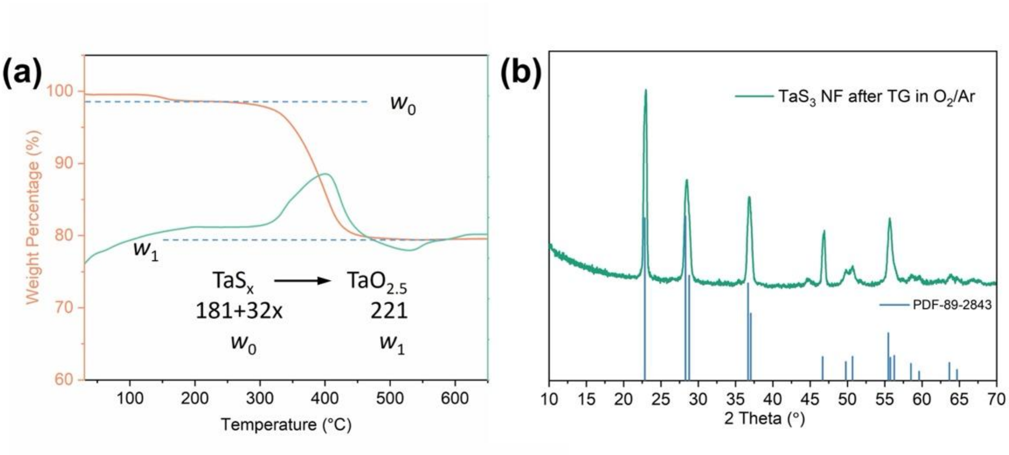

Figure S 8. (a) TG-DSC thermograms for a TaS_3 NF film in heated air atmosphere at a rate of 10 °C min $^{-1}$, showing the weight percentage and heat flow curves. The small initial weight loss at *ca*. 150 °C may be attributed to the removal of absorbed water. (b) PXRD of TaS_3 NFs after TG experiment which shows the complete conversion of TaS_3 to Ta_2O_5 (single phase).

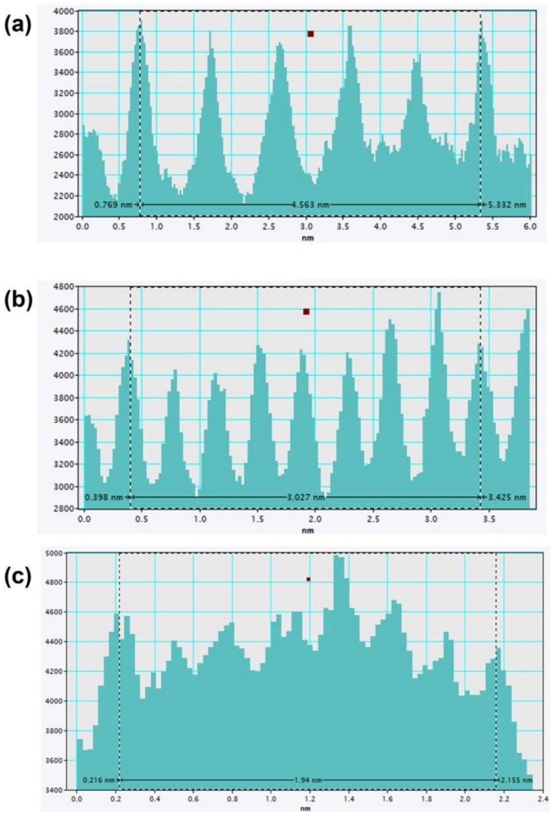

Figure S 9. Lattice fringe spacing measurements for: (a) the (200) and (b) (20-4) crystal planes of the TaS_3 NF film, and (c) the (002) plane of monoclinic Ta_2O_5, as taken from the HRTEM image.

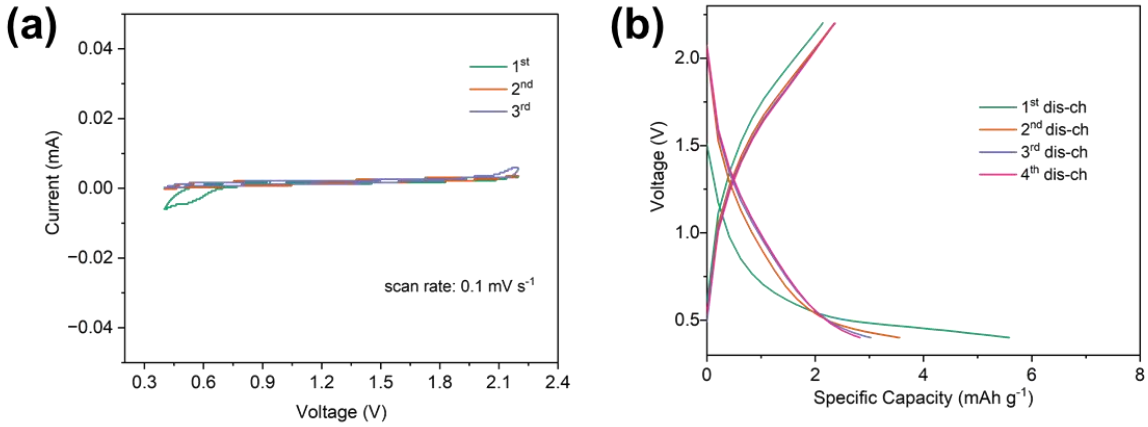

Figure S 10. (a) CV curves (scan rate of 0.1 mV s^{-1} between 0.4 V and 2.2 V) and (b) the initial four (dis)charge curves (50 mA g^{-1}) of a TaS_3 NF film electrode in a pure APC electrolyte.

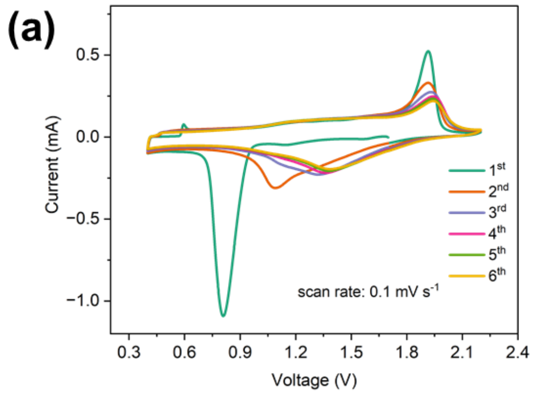

Figure S 11. Initial six CV curves of: (a) the bulk TaS_3 electrode in a MLIB cell and (b) the TaS_3 NF film electrode in a LIB cell at a scan rate of 0.1 mV^{-1}.

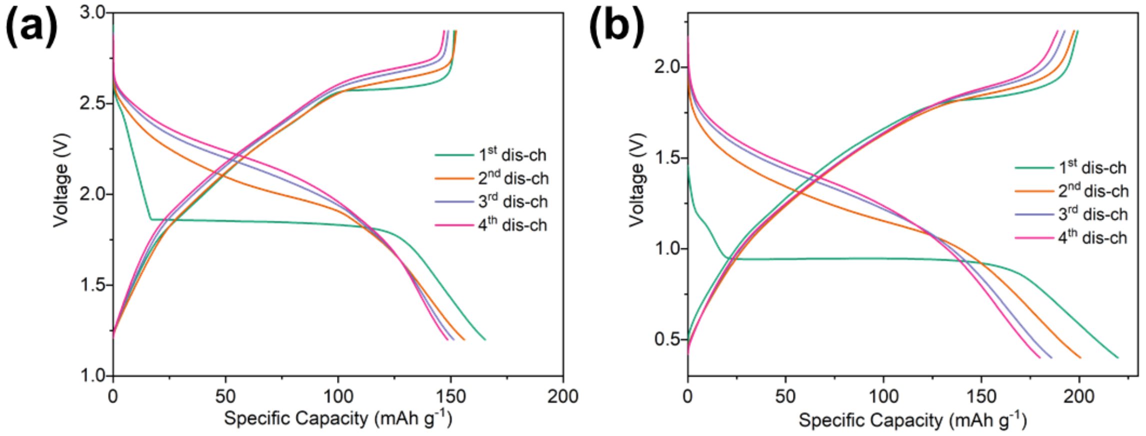

Figure S 12. Initial four (dis)charge curves of: (a) the TaS_3 NF film electrode in an LIB cell (1.2 V - 2.9 V) and (b) the bulk TaS_3 electrode in an MLIB cell (0.4 V - 2.2 V) at a current density of 50 mA g^{-1}.

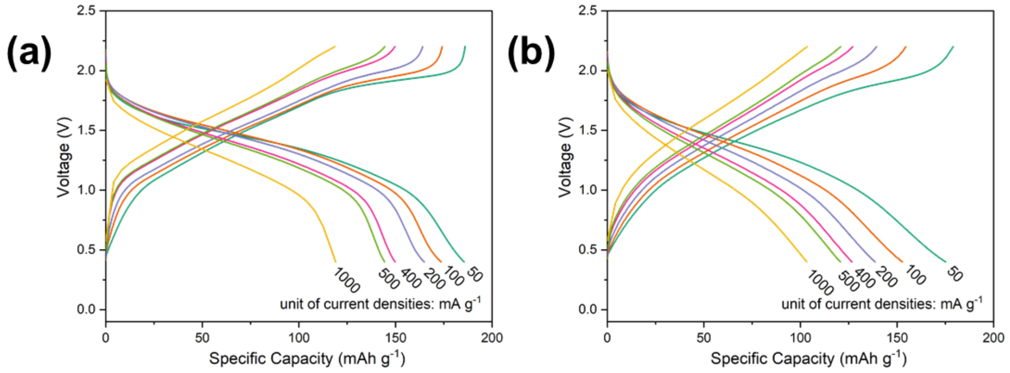

Figure S 13. Selected (dis)charge curves of: (a) the TaS_3 NF film electrode and (b) the bulk TaS_3 electrode, in MLIB cells at current densities of 50 mA g^{-1}, 100 mA g^{-1}, 200 mA g^{-1}, 400 mA g^{-1}, 500 mA g^{-1}, and 1000 mA g^{-1}.

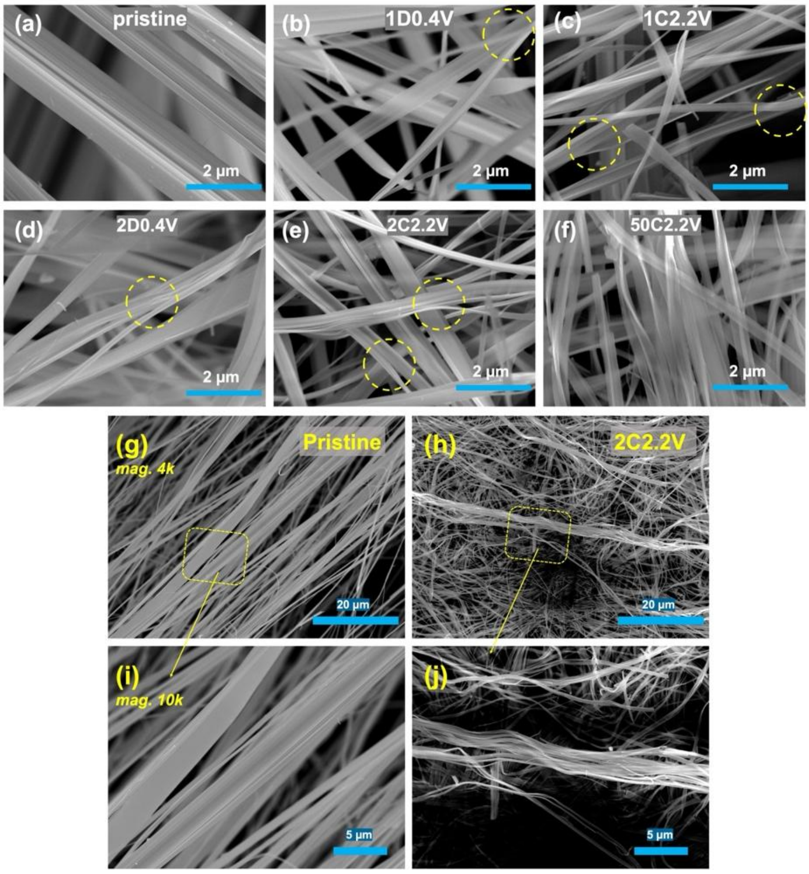

Figure S 14. Higher-magnification SEM images of TaS_3 NF at the (a) uncharged, pristine state, and after the (b) 1D0.4V and (c) 1C2.2V, (d) the 2D0.4V and (e) 2C2.2V, and (f) the 50C2.2V states. A comparison of (g, i) pristine and (h, j) 2C2.2V-state, representative

exfoliated large TaS_3 fibres. The yellow dashed circles indicate the regions where (partial) peeling occurs.

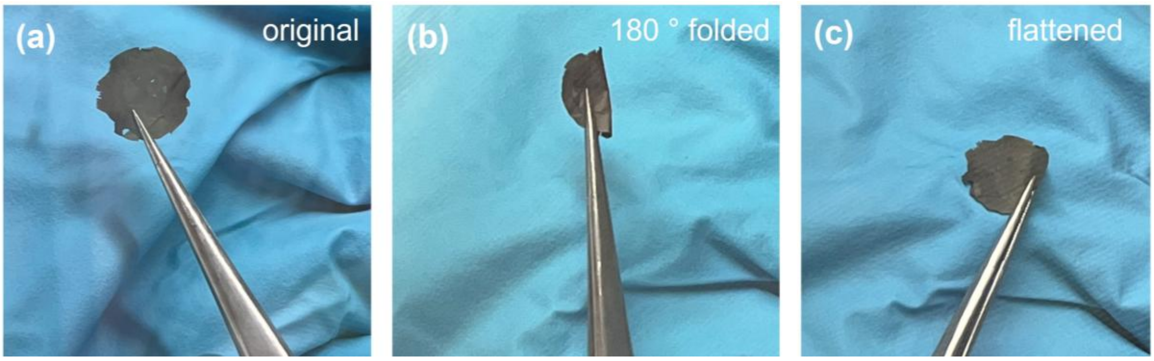

Figure S 15. Photographs of the TaS_3 NF film electrode after the 50th charge: (a) as collected removed from the cell, (b) subsequently folded 180°, and (c) once recovered from folding.

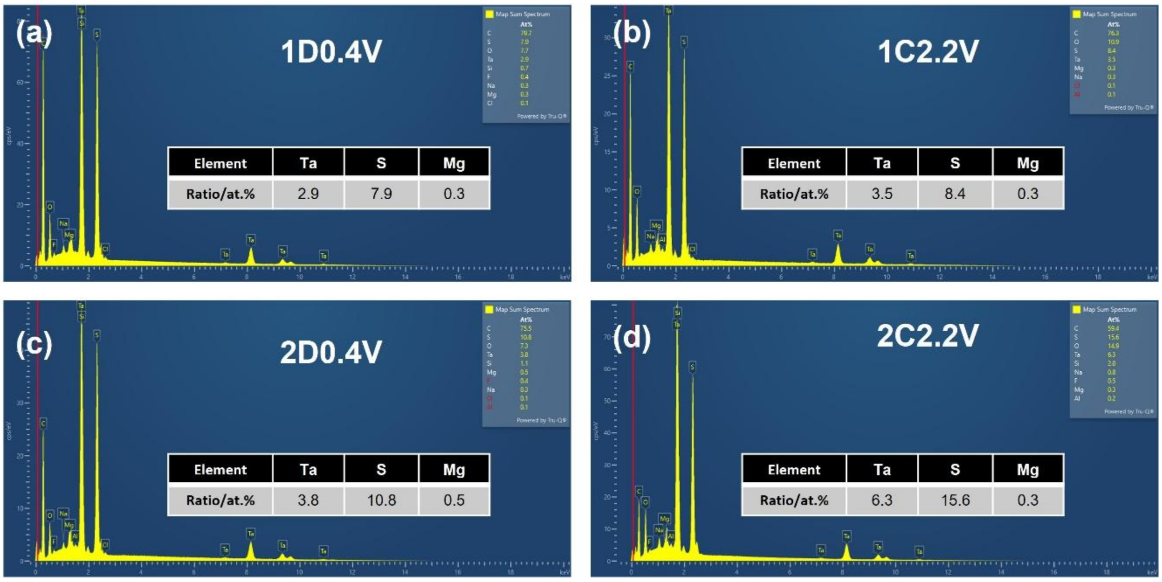

Figure S 16. EDS spectra of TaS_3 NF film electrodes in the (a) first discharged (1D0.4V), (b) first charged (1C2.2V), (c) second discharged (2D0.4V), and (d) second charged (2C2.2V) states in MLIBs.

Table S 5. Normalised Ta, S, and Mg EDS results (from probed data in **Figure S15**).

State	Atomic percentage (at.%)			Mg/Ta ratio
	Ta	S	Mg	
1D0.4V	26.13	71.17	2.70	0.10
1C2.2V	28.69	68.85	2.46	0.09
2D0.4V	25.17	71.52	3.31	0.13
2C2.2V	28.38	70.27	1.35	0.05

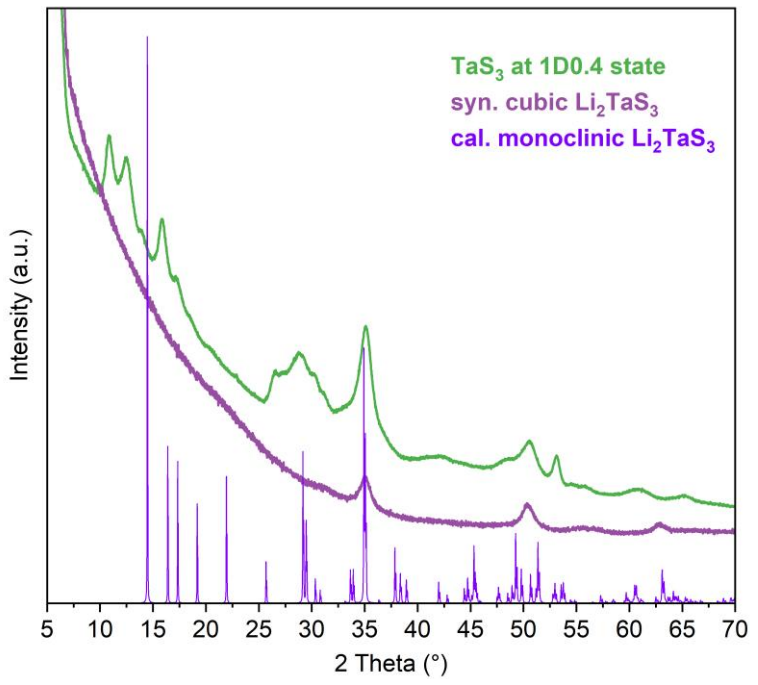

Figure S 17. Transmission PXRD pattern of the first discharged TaS_3 NF electrode and the mechanically synthesised cubic Li_2TaS_3, as well as a simulated monoclinic Li_2TaS_3 PXRD based on monoclinic Li_2NbS_3.

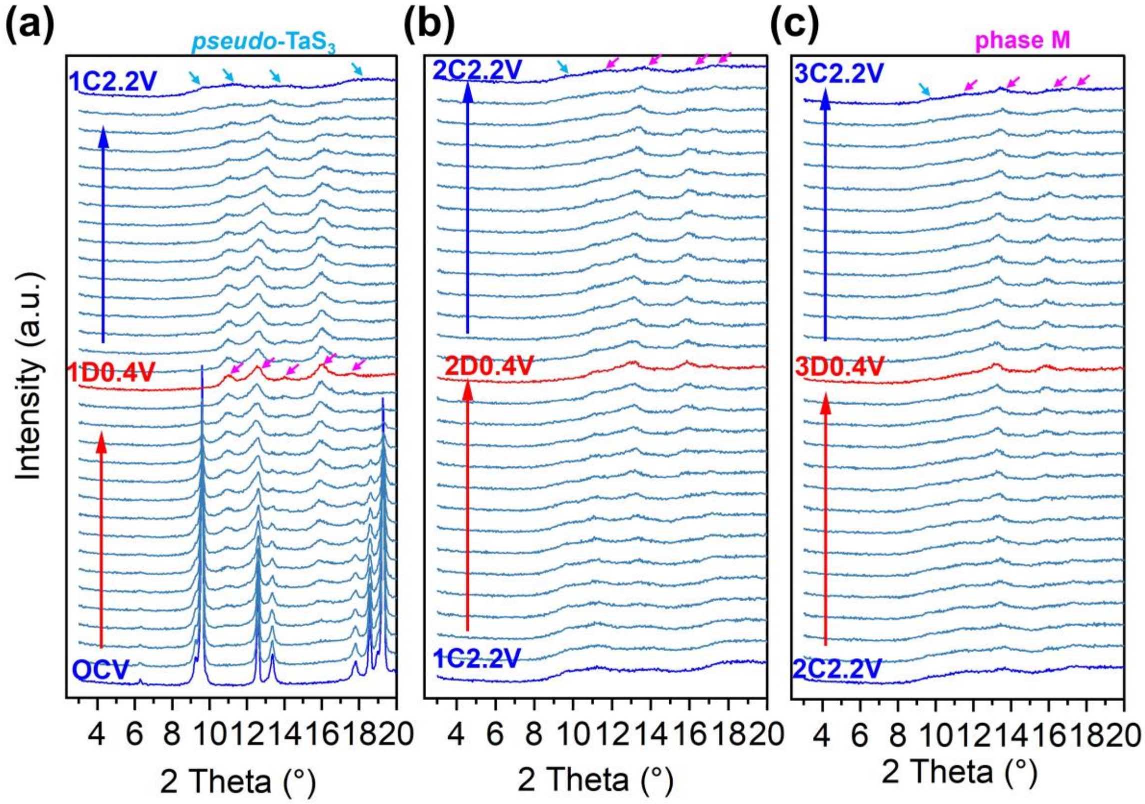

Figure S 18. Stack plots of *operando* PXRD patterns of the TaS_3 NF electrode: (a) the first, (b) the second, and (c) the third discharge and charge cycles. Long red and deep blue arrows indicate discharging and charging process, respectively. Short pink and blue arrows represent phase M and *pseudo*-TaS_3, respectively.

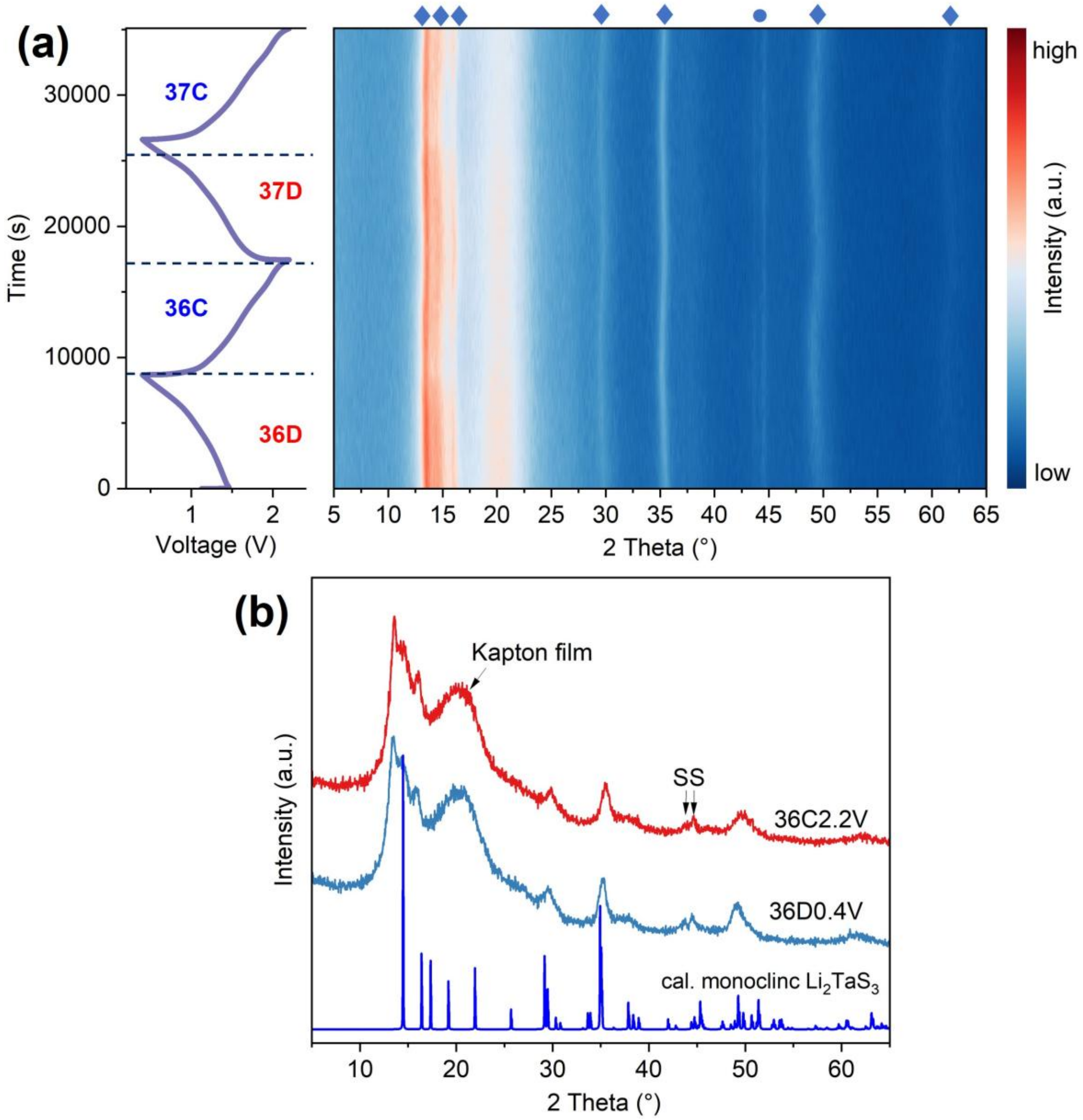

Figure S 19. (a) The 36th and 37th discharge-charge curves and their contour plots of the corresponding *operando* PXRD patterns for the TaS_3 NF film electrode. Note that the blue rhombuses and circle represent the diffraction peaks attributed to phase M and stainless steel cell, respectively. (b) The patterns of the electrode at 36D0.4V and 36C2.2V states taken from the above *operando* data, compared the PXRD pattern of monoclinic Li_2TaS_3. Peaks for the Kapton film and stainless steel have been marked in the figure.

The CV peak current can be expressed as a power law in Equation S1:[3]

$$i = av^b \quad \text{Equation S1}$$

in which i is the current (mA), v is the scan rate (mV s^{-1}), and a and b are adjustable constants. For pure diffusion-mediated processes, i values are proportional to $v^{1/2}$ (b = 0.5), while for capacitative processes, i values are proportional to v (b = 1). In mixed processes, b values typically range from 0.5 to 1.0. By performing a linear fit of log(i) against log(v) (Equation S2), one can gain a general understanding of the types of charge storage phenomena in the electrochemical reactions.

$$log(i) = log(a) + blog(v) \quad \text{Equation S2}$$

The current sum of these behaviours at a voltage value in CV curves can be described using Equation S3, allowing for the determination of the weights of surface-confined capacitative (k_1) and bulk diffusion-controlled (k_2) phenomena by linear fitting of $i/v^{1/2}$ against $v^{1/2}$.[4]

$$i = k_1 v + k_2 v^{1/2} \text{ or } \frac{i}{v^{1/2}} = k_1 v^{1/2} + k_2 \quad \text{Equation S3}$$

The diffusion coefficient can be determined according to the Equation S4 according to Fick's law:[5]

$$D = \frac{4}{\pi\tau}\left(\frac{m_B V_M}{M_B S}\right)^2 \left(\frac{\Delta E_s}{\Delta E_\tau}\right)^2 \quad \text{Equation S4}$$

where τ, m_B, V_M, and M_B, and S stand for constant current pulse duration (s), mass (g), molar volume (cm^3 mol^{-1}), molar mass (g mol^{-1}), and contact area (cm^{-2}) of the TaS_3 NF electrode material, respectively. ΔE_τ is the potential change during the current pulse without *IR* drop (vertical potential drop/rise while initiating/stopping the current pulse due to ohmic and charge transfer resistances),[6, 7] and ΔE_s represents the potential gap between the equilibrium states before and after the current pulse, both of which could be collected from the as-measured GITT curves (Figure 8d).

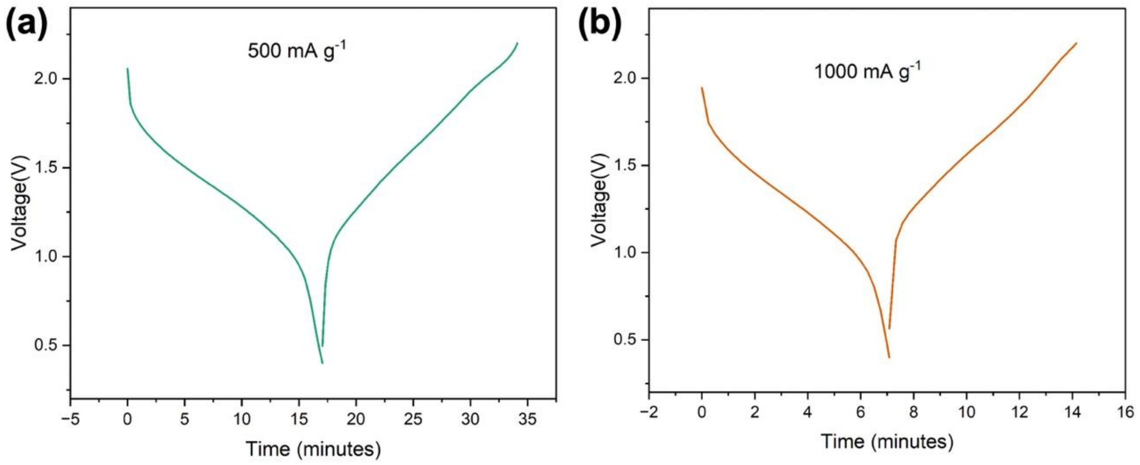

Figure S 20. Voltage-time curves of the TaS_3 NF at (a) 500 mA g^{-1} and (b) 1000 mA g^{-1}.

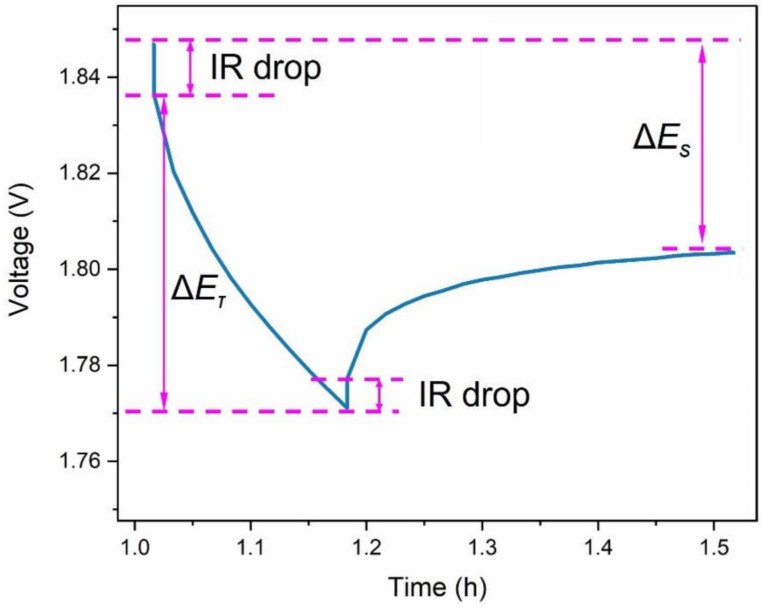

Figure S 21. A single current pulse and relaxation pair taken from the GITT curve of the TaS_3 NF film electrode shown in Figure 8d showing ΔE_τ, ΔE_s, and *IR* drop.

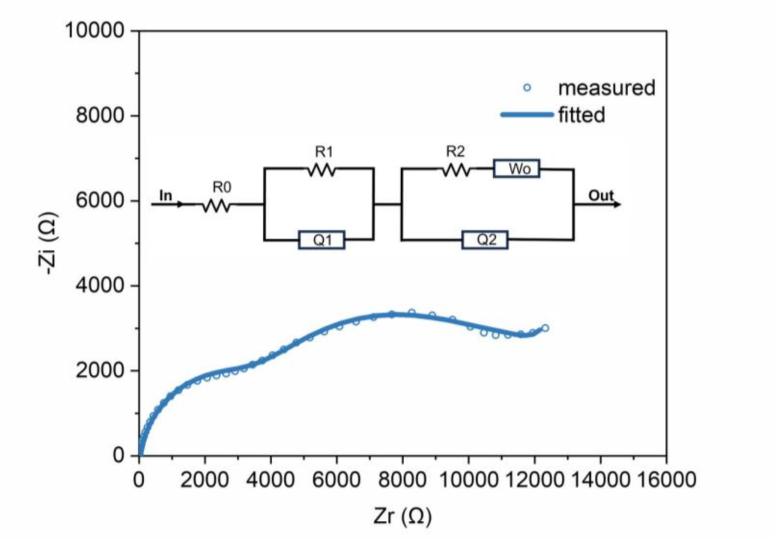

Figure S 22. Measured (circles) and fitted (solid line) Nyquist plot and corresponding equivalent circuit fit to the EIS data for the (-)Mg|LiAPC|TaS_3 NF film electrode(+) cell before cycling. The fitted equivalent circuit is presented as an inset in the graph.

Table S 6. Detailed values of parameters in the obtained equivalent circuit model for the three (-)Mg|LiAPC|TaS_3 NFs(+) cells at uncycled, the first, and 20th charged states.

		$Q_1(s^{\alpha}\ \Omega^{-1})$	$Q_2(s^{\alpha}\ \Omega^{-1})$	$R_0(\Omega)$	$R_1(\Omega)$	$R_2(\Omega)$	$W_0(\Omega\ s^{-0.5})$
before cycle	value	1.760E-05	1.220E-04	7.495	3296.350	7998.660	793.684
	error	1.130E-08	1.320E-07	0.147	3.490	1.916	0.667
1st charge	value	2.880E-06	2.020E-04	11.812	20.782	742.172	310.169
	error	1.840E-14	3.660E-06	0.174	0.417	20.711	5.556
20th charge	value	4.920E-06	2.260E-04	12.177	54.674	229.675	146.071
	error	9.330E-14	1.100E-05	0.898	3.698	14.338	6.526

Note S1: On the Limitations of Slurry-Cast Processing of TaS_3 NF for Electrode Comparison

In our study, TaS_3 NF exhibits flexible, centimetre-scale long fibrous morphology. This renders the film mechanically resistant to grinding or ball-milling; attempts to prepare a fine powder suitable for slurry casting proved unsuccessful under our experimental conditions.

As such, a direct performance comparison between TaS_3 NF-based free-standing and slurry-cast electrodes using the same material was not experimentally feasible. To address this, we employed bulk TaS_3 with similar crystallographic features to fabricate slurry-cast electrodes as a practical alternative. Despite this limitation, the comparison (Figure 3) still provides meaningful insight into the electrochemical benefits of the free-standing fibrous configuration for this class of redox-active trichalcogenide materials.

References

(1) Mayorga-Martinez, C. C.; Sofer, Z.; Luxa, J.; Huber, Š.; Sedmidubský, D.; Brázda, P.; Palatinus, L.; Mikulics, M.; Lazar, P.; Medlín, R.; et al. TaS_3 Nanofibers: Layered Trichalcogenide for High-Performance Electronic and Sensing Devices. *ACS Nano* **2018**, *12* (1), 464-473. DOI: https://doi.org/10.1021/acsnano.7b06853.

(2) Meerschaut, A.; Guemas, L.; Rouxel, J. Structure and properties of the new phase of the pseudo one-dimensional compound TaS_3. *Journal of Solid State Chemistry* **1981**, *36* (1), 118-123. DOI: https://doi.org/10.1016/0022-4596(81)90199-7.

(3) Ren, W.; Zhang, H.; Guan, C.; Cheng, C. Ultrathin MoS_2 Nanosheets@Metal Organic Framework-Derived N-Doped Carbon Nanowall Arrays as Sodium Ion Battery Anode with Superior Cycling Life and Rate Capability. *Advanced Functional Materials* **2017**, *27* (32), 1702116. DOI: https://doi.org/10.1002/adfm.201702116.

(4) Wang, J.; Polleux, J.; Lim, J.; Dunn, B. Pseudocapacitive Contributions to Electrochemical Energy Storage in TiO_2 (Anatase) Nanoparticles. *The Journal of Physical Chemistry C* **2007**, *111* (40), 14925-14931. DOI: https://doi.org/10.1021/jp074464w.

(5) Wen, C. J.; Boukamp, B. A.; Huggins, R. A.; Weppner, W. Thermodynamic and Mass Transport Properties of " LiAl " *Journal of The Electrochemical Society* **1979**, *126* (12), 2258. DOI: https://doi.org/10.1149/1.2128939.

(6) Knehr, K. W.; Biswas, S.; Steingart, D. A. Quantification of the Voltage Losses in the Minimal Architecture Zinc-Bromine Battery Using GITT and EIS. *Journal of The Electrochemical Society* **2017**, *164* (13), A3101. DOI: https://doi.org/10.1149/2.0821713jes.

(7) Jing, P.; Stevenson, S.; Lu, H.; Ren, P.; Abrahams, I.; Gregory, D. H. Pillared Vanadium Molybdenum Disulfide Nanosheets: Toward High-Performance Cathodes for Magnesium-Ion Batteries. *ACS Applied Materials & Interfaces* **2023**, *15* (44), 51036-51049. DOI: https://doi.org/10.1021/acsami.3c10287.
